# Homeostatic Counter‐Regulation Mediates Spermidine‐Induced Triacylglyceride Reduction in *Drosophila melanogaster*—From Phenotype to Molecular Mechanism

**DOI:** 10.1096/fj.202502620R

**Published:** 2025-10-21

**Authors:** Kai Lüersen, Celina Runke, Bernhard Blank‐Landeshammer, Julian Weghuber, Ronald P. Kühnlein, Thomas Roeder, Gerald Rimbach

**Affiliations:** ^1^ Division of Food Science, Institute of Human Nutrition and Food Science University of Kiel Kiel Germany; ^2^ Center of Excellence Food Technology and Nutrition University of Applied Sciences Upper Austria Wels Austria; ^3^ FFoQSI–Austrian Competence Centre for Feed and Food Quality, Safety & Innovation Tulln Austria; ^4^ Institute of Molecular Biosciences, NAWI Graz University of Graz Graz Austria; ^5^ Field of Excellence BioHealth University of Graz Graz Austria; ^6^ Division of Molecular Physiology, Institute of Zoology University of Kiel Kiel Germany

**Keywords:** adipokinetic hormone, dietary protein, obesity, polyamine catabolism, polyamine excretion

## Abstract

The polyamines putrescine, spermidine (Spd), and spermine have essential functions in cell growth and proliferation. Previous research has unveiled a potential link between polyamine metabolism and triacylglyceride (TAG) homeostasis. In this study, we utilized *Drosophila melanogaster* as a model to study the impact of dietary (Spd) on body TAG stores. We found that food supplementation with 1.0 mM and 2.5 mM Spd prevented the build‐up of TAG stores in female fruit flies monitored for up to 14 days post‐eclosion in a dose‐ and time‐dependent manner, without affecting their total protein content. Notably, a 7‐day treatment with 2.5 mM Spd also counteracted high‐sugar diet–induced obesity and accelerated the breakdown of existing TAG reserves in obese females. Mechanistic analysis revealed that the adipokinetic hormone (Akh) pathway but not the TAG lipase Brummer (Bmm) was required for the TAG‐lowering bioactivity of Spd. Remarkably, this TAG‐reducing activity was completely abolished when flies were fed a high‐yeast diet, which increases dietary protein. Analyses of the polyamine pattern of flies and their excreta revealed that under low dietary yeast conditions, the administered Spd did not result in a rise in the endogenous Spd level. Instead, the Spd metabolites putrescine and N^1^‐acetylated Spd were increased, which provides evidence for the induction of the catabolic arm of the polyamine pathway. Together, our data suggest that the administration of Spd at lower mM concentrations, if combined with a low‐protein diet, stimulates Akh‐dependent catabolic processes that facilitate the reduction of fat stores in *D. melanogaster* . This work uncovers a diet‐dependent metabolic role of Spd in fat storage regulation, which awaits confirmation in mammals and humans, and highlights the importance of nutrient context in modulating polyamine‐mediated metabolic outcomes.

## Graphical abstract

In *Drosophila melanogaster* females fed a standard diet containing 2.5% yeast extract (low‐yeast diet), spermidine (Spd) administration does not enhance the endogenous Spd pool but increases the excretion of catabolic N^1^‐acetylspermidine (N1AcSpd) and reduces fat stores. This process requires the catabolic Akh/AkhR pathway. Conversely, if the yeast extract concentration in the standard diet is raised to 10% (high‐yeast diet), the fat‐reducing impact of dietary Spd is abolished, the endogenous Spd level is increased, and the excretion of N1AcSpd is diminished.

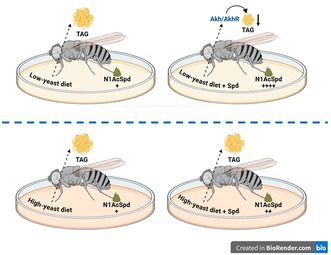

## 1 Introduction

Dysregulation of lipid homeostasis is associated with numerous diseases, with obesity representing one of the most widespread health issues globally [1, 2, 3]. Obesity is characterized by excess fat accumulation caused by an imbalance between energy intake and energy expenditure, often due to excessive calorie intake or endocrine dysfunction. It is a major risk factor for cardiometabolic diseases such as metabolic syndrome, coronary heart disease, and type 2 diabetes. Therefore, nutritional factors that beneficially modulate lipid metabolism are of particular interest.

The fruit fly *Drosophila melanogaster* is a well‐established model organism for nutritional phenotyping [4, 5, 6], including the investigation of nutrient effects on the evolutionarily highly conserved energy metabolism [7, 8]. In *Drosophila*, as in most animals, triacylglycerides (TAG) are the main energy store, primarily accumulated in lipid droplets of the fat body, an organ functionally equivalent to both vertebrate liver and white adipose tissue [7, 9]. Smaller TAG pools exist in the intestine, oenocytes, ovaries, and brain [10]. Energy‐demanding processes such as egg production and embryogenesis rely on these fat stores [11, 12, 13, 14]. Dietary interventions demonstrate that TAG levels in fruit flies depend on the nutritional status: starvation depletes fat stores enabling survival [14, 15], while chronic high‐sugar feeding promotes fatty acid and TAG synthesis, yielding an obese phenotype reminiscent of mammalian metabolic syndrome [9, 16]. Key enzymes governing TAG synthesis and breakdown are evolutionarily conserved [7, 9, 17]. Likewise, the endocrine control of lipid metabolism resembles the vertebrate insulin/glucagon systems [14, 17]. *Drosophila* Insulin‐like peptides (dIlp) promote the buildup of fat depots after sugar intake [17], whereas a low energy status stimulates the release of a functional glucagon analog called adipokinetic hormone (Akh) from neuroendocrine cells of the corpora cardiaca [15]. Akh activates both cAMP‐ and inositol‐1,4,5‐trisphosphate/Ca^2+^‐dependent signaling cascades via its cognate G‐protein‐coupled receptor (Adipokinetic hormone receptor; AkhR), mobilizing fat stores in fat body cells, with the downstream effector TAG lipase(s) remaining elusive [9, 14, 18]. The Brummer (Bmm) lipase, a homologue of mammalian adipose triglyceride lipase (ATGL), cooperates with the Akh pathway in TAG mobilization. Bmm expression is regulated, at least in part, by Forkhead box, sub‐group O (Foxo) dependent transcription downstream of the Akh/cAMP/protein kinase A pathway [14, 19]. Consistent with their physiological functions, *Drosophila Akh*, *AkhR*, and *bmm* loss‐of‐function mutants are obese [18, 19].

Spermidine (Spd), a ubiquitous polyamine, participates in many fundamental biological processes, including gene regulation, proliferation, growth, differentiation, and apoptosis [20, 21]. Its polycationic character allows interaction with a wide spectrum of negatively charged macromolecules including RNA, DNA, phospholipids, and proteins. As both deficiency and excess are detrimental, intracellular Spd levels are tightly regulated by complex anabolic and catabolic interconversion pathways [22]. Spd biosynthesis requires ornithine and S‐adenosylmethionine (SAM). Both metabolites are decarboxylated by ornithine decarboxylase (ODC) and SAM decarboxylase (SAMDC), respectively, leading to putrescine and decarboxylated SAM, dcSAM. Spd synthase then transfers an aminopropyl group from dcSAM to putrescine. Further elongation of Spd by a similar second dcSAM transfer catalyzed by spermine synthase yields spermine. Besides biosynthesis, exogenous polyamine sources—diet and intestinal microbiota—contribute to the Spd pool [23, 24]. In the human diet, cereals, legumes, soybeans, meat, and fermented foods such as cheese are primary sources of Spd [25, 26]. Average daily Spd intake, assessed for numerous industrialized countries, ranges from 33 μmol in Turkey to 103 μmol in Spain [25]. The catabolic pathway counteracting a surplus of intracellular Spd has so far been best studied in mammals and involves a spermine/spermidine N^1^‐acetyltransferase (SSAT) and a polyamine oxidase (PAO). N^1^‐acetylated Spd can be excreted or cleaved back to putrescine by PAO [27].

Transgenic mouse models with a modified interconversion pathway unveiled a link between polyamine and energy metabolism [28, 29, 30]. Moreover, Spd administration improves lipid metabolism and reduces body fat stores in mice [31, 32, 33, 34, 35, 36, 37], consistent with the proposed caloric restriction mimetic properties of Spd [1]. Epidemiological data likewise revealed an inverse correlation between dietary Spd intake and obesity‐associated traits in humans [38].

Similar to mammals, polyamine synthesis of *D. melanogaster* includes homologues of ODC, SAMDC, Spd synthase, and spermine synthase [39, 40]. However, genes involved in fly Spd catabolism await characterization, although putative homologues have been suggested [41]. Notably, genetic models of polyamine synthesis exhibit altered lipid stores [39, 40]. Yet, the influence of dietary Spd on energy metabolism in *Drosophila* has remained unexplored.

In this study, we investigated the impact of dietary Spd supplementation on the polyamine patterns and body composition in adult fruit flies and analyzed associated phenotypic consequences. Spd administration enhanced polyamine catabolism, as evidenced by the elevated production and excretion of putrescine and N^1^‐acetylspermidine (N^1^‐acetyl‐Spd). This was associated with profound reductions in energy stores, resilience towards starvation, and egg production. Additionally, Spd counteracted obesity induced by high‐sugar feeding. By employing null mutants, we found that Akh signaling is required for Spd‐induced TAG decline. This metabolic impact of Spd was strongly dependent on the composition of the *Drosophila* diet, with the proportion of yeast being pivotal. Our data suggest that Spd exerts caloric restriction mimetic effects that promote the reduction of fat stores.

## 2 Methods

### 2.1 Fly Strains, Cultivation and Nutrient Media

The *w* ^*1118*^ (RRID:BDSC_3605) strain and the Oregon‐RC (RRID:BDSC_5) wild‐type strain were obtained from the Bloomington *Drosophila* Stock Center (Indiana University, Bloomington, Indiana, USA). In addition, we used the mutant strains *Akh* ^*attP*^ (RRID:BDSC_84448), *AkhR* ^*attP*^ (RRID:BDSC_84449), and *bmm* ^*1*^ [19]. All flies were kept at 25°C, 60% humidity, and a 12 h/12 h day/night cycle (climate cabinets HPP750 and HPP110, respectively; Memmert, Schwabach, Germany).

Flies were maintained on standard Caltech medium (CT_2.5_, the subscripts indicate the percentage of inactive yeast extract in the respective media) containing 1.0% agar (Kobe I, Carl Roth, Karlsruhe Germany), 5.5% glucose (Carl Roth), 3.0% sucrose (Carl Roth), 6.0% cornmeal (Genesee Scientific, San Diego, USA), 2.5% inactive dry yeast (Genesee Scientific), and 0.2% tegosept (Genesee Scientific) and 0.3% propionic acid (Carl Roth) as preservatives [5]. The inactive yeast extract serves as the main protein source in our *Drosophila* diets, but due to its complex composition, it also provides fiber, minerals, and vitamins, as well as small amounts of lipids and carbohydrates [42, 43]. To examine the impact of food composition, various experimental fruit fly diets were used. There is currently no standard *Drosophila* diet [43], which is why a variety of different media are used in fruit fly feeding studies, with protein content typically varying between 2.5% and 10%. A CT_10_ medium was generated by increasing the yeast content of the CT_2.5_ diet from 2.5% to 10%. The standard sucrose‐yeast medium (SY_10_) consisted of 5% sucrose, 10% inactive dry yeast, 2% agar, 0.2% tegosept, and 0.3% propionic acid [5]. A SY_2.5_ version of this medium contained a reduced proportion of inactive dry yeast of 2.5%. The cornmeal‐glucose (CG_2.5_) diet [44] contained 6.0% cornmeal, 5.0% glucose, 2.5% inactive dry yeast, 0.8% agar, 0.2% tegosept, and 0.3% propionic acid. As described in [44], a diluted cornmeal‐glucose food (CG_DR_ medium), which was employed in nutrient restriction experiments, was prepared by reducing the content of cornmeal, glucose, and inactive dry yeast by 50%.

High‐glucose diets were prepared by increasing the glucose content of the CG_2.5_ diet from 5.0% to 20% and 30% glucose, respectively. In addition, similar control and high‐sugar diets (HSD) were generated by replacing glucose with sucrose and fructose, respectively. For all diet intervention studies, fruit flies were randomly allocated to the different experimental media. The stage, sex, and age of the fruit flies examined are indicated either in the respective method section or in the corresponding legends of the figures and tables.

For Spd‐supplementation studies, an aqueous stock solution with a concentration of 0.5 M Spd (Catalogue #132740250, Thermo Scientific, Darmstadt, Germany) was prepared. Spd was added to the different *Drosophila* media at a final concentration of 1.0 or 2.5 mM after autoclaving, when the medium had cooled to approximately 65°C. These concentrations correspond to those used in previous *D. melanogaster* studies, in which positive effects of Spd administration on aging were determined [45, 46].

### 2.2 Determination of Food Intake and Collection of Excreta Using the Excretion‐Quantification (Ex‐Q) Assay

Food intake of flies was assessed with the excretion‐quantification (Ex‐Q) assay according to [47]. Groups of 12 freshly eclosed females and 12 males were kept on experimental media for 3 days. On Day 3, 20 female flies were transferred to Ex‐Q vials with a lid‐in‐lid construction. The inner microtube lids (of 1.5 mL Eppendorf tubes) were filled with experimental diets dyed with 0.5% (w/v) erioglaucine (Carl Roth). The flies remained in these Ex‐Q tubes for 24 h before they were removed and their excreta were rinsed off the tube walls and outer lids with 1 mL distilled water. For the photometric measurement, the absorbance of the samples was determined at 620 nm. Moreover, 500 μL of the remaining excreta solution were used for polyamine determination by HPLC (see below).

### 2.3 Determination of Body Composition and Body Weight

Except stated otherwise, for experiments involving the assessment of body weight and body composition, groups of freshly eclosed male and female flies (randomly assigned to groups of 12 per sex and vial) were pre‐fed on treatment media for 7 days, before they were transferred to empty vials for 1 h for gut cleansing. The flies were then separated by sex, and their fresh body weight was determined in groups of 12 flies each on a precision scale. For storage, the animals were immediately frozen at −80°C. To determine the difference between fresh and dry weights, the fruit flies, separated by sex, were first weighed in groups of 100 individuals immediately after harvesting using a precision scale. The flies were then frozen at −80°C for 15 min, before being placed in a drying oven at 65°C. After 24 h, the dry masses were measured. Additionally, migrating third‐instar larvae that had left the medium were harvested in groups of 10 individuals for body weight and body composition determination. For this, they were removed from the vial walls using feather tweezers, briefly washed in water to remove the medium, and dried on absorbent paper. After weighing, they were frozen at −80°C.

To assess body composition parameters, 10 flies per treatment sample were lysed for 6 min in 500 μL phosphate buffer saline containing 0.05% Triton X‐100 (PBST) using a TissueLyzer (Qiagen, Hilden, Germany). Subsequently, the fly lysates were centrifuged at 10000×*g* for 10 min at 4°C. Aliquots of the supernatants were incubated at 70°C for 5 min before they were immediately used for glucose measurement with the DIALAB glucose GPO‐PAP test kit (DIALAB, Wr. Neudorf, Austria). To determine the glycogen content, 30 μL aliquots of the samples were first digested with 30 μL amyloglucosidase (0.5 U/mL; Megazyme Ltd., Wicklow, Ireland) for 24 h at 37°C before they were analyzed with the DIALAB glucose GPO‐PAP test kit.

To assess the protein and TAG content, the supernatants of the lysate samples were diluted 1:3 with PBST. The diluted samples were used directly for spectrophotometric protein determination with the Pierce BCA protein assay kit according to the manufacturer's instructions (Thermo Fisher Scientific, Darmstadt, Germany). For TAG measurement, a 100 μL aliquot of the sample was filled into new tubes and immediately placed in a heating block at 70°C for 5 min. The TAG concentration of the fly lysates was determined spectrophotometrically using the Triglyceride‐GPO‐PAP test kit according to the manufacturer's instructions (DIALAB).

In addition, the TAG contents of fruit flies were exemplarily analyzed by thin layer chromatography (TLC) according to [48]. Ten flies of each treatment group were homogenized in 250 μL chloroform/methanol (2:1; v/v) (TissueLyzer) and left for 2 h at room temperature on a rotator. Subsequently, the debris was spun down at 14,000×*g* for 5 min. 5 μL of the supernatants were loaded on a POLYGRAM SIL G TLC plate (20 × 20 cm; Macherey‐Nagel, Düren, Germany). Lard (5–50 μg) was used as a standard. After separation that was carried out by using the mobile phase n‐hexane/ethylether (4:1; v/v), the plates were dried and then stained with an oxidative ceric ammonium molybdate (CAM) solution (2.5 g ammonium heptamolybdate tetrahydrate (Sigma), 1 g of cerium (IV) sulfate hydrate complex with sulfuric acid (Sigma), in 90 mL H_2_O and 10 mL concentrated H_2_SO_4_). TAG signals were developed by a 20 min incubation at 80°C before a photo was taken.

### 2.4 Polyamine Determination by Reverse‐Phased HPLC

The polyamine pattern of fruit flies and their excreta were assessed by following the sample preparation and HPLC separation methods described in Kabra et al. [49] and Heinick et al. [50]. Briefly, 10–12 female fruit flies (age 7 days) were transferred to empty vials for 1 h to allow gut cleansing, before being lysed in 1.0 mL 5% TCA containing 2.0 μg/mL of the internal standard diaminoheptane (Sigma) using a TissueLyser (10 min at 25 Hz). After centrifugation for 20 min at 14000×*g* and 4°C, 500 μL of the supernatant was transferred to a new Eppendorf tube. For excreta samples, 450 μL of the excreta solution (see Ex‐Q assay above) were mixed with 50 μL 50% TCA containing 1 μg diaminoheptane. To these fruit fly and excreta samples, respectively, 250 μL of a saturated NaHCO_3_ and an extra spatula tip of solid NaHCO_3_ were given, before 500 μL dansyl chloride (7 mg/mL acetone) were added. Derivatization was carried out in the dark in a water bath at 70°C for 10 min. To eliminate excess dansyl chloride, 250 μL of a 100 mg/mL proline solution was added and the samples were incubated for another 3 min at 70°C. Subsequently, the dansylated polyamines were extracted twice with 500 μL ethyl acetate. After evaporation of the solvent in a SpeedVac (SPD111V‐230; Thermo Scientific, Langenselbold, Germany), the pellets were resuspended in 100 μL acetonitrile.

For HPLC analyses, 20 μL of each sample were injected and the dansylated polyamines were separated on a nucleosil 5 μm C18 column (250 × 4.6 mm, Phenomenex, Aschaffenburg, Germany) using the gradient listed in Table S1. In order to record the complete polyamine spectrum, dansylated polyamines were detected by UV absorption at *λ* = 254 nm (DAD, Agilent Technologies, Waldbronn, Germany) and simultaneously by more sensitive fluorescence detection (FD, Agilent) (excitation wavelength 365 nm, emission wavelength 480 nm). Polyamine standards (cadaverine, N‐acetylputrescine, N‐acetylspermine, putrescine, Spd, spermine (Sigma), N^1^‐acetylspermidine (Cayman chemicals, Ann Arbor, Michigan, USA), and N^8^‐acetylspermidine (MCE MedChemExpress, Monmouth Junction, United States)) were used to generate calibration curves.

### 2.5 Egg Laying Assay

Twelve freshly eclosed male and 12 female flies were randomly distributed to each of the different experimental media. On Day 3, the males were discarded and two mated females were placed on each fresh experimental medium. Subsequently, the eggs laid on Days 4, 5, and 8 were counted.

### 2.6 Determination of Spontaneous Locomotor Activity

Spontaneous locomotory activity was quantified using the *Drosophila* Activity Monitoring (DAM) system (TriKinetics Inc., Princeton, MA, USA). Freshly eclosed males and females were randomly placed on each of the different experimental media on Day 1. On Day 4, the males were discarded and 20 females each were placed in a tube with the appropriate treatment medium. On Day 5, the females were transferred to glass tubes containing the respective treatment food. These glass tubes were then placed in the activity monitor and flies were maintained under standard culture conditions. For the second day in the DAM system, the mean locomotor activity of each vial was calculated by dividing the total number of beam brakes registered within the 24 h period by the respective number of flies per vial. Additionally, this calculation was performed separately for the day (06:00 a.m. to 06:00 p.m.) and night phase (06:00 p.m. to 06:00 a.m.).

### 2.7 Starvation Stress Assay

Freshly eclosed flies were randomly sorted into groups of 12 males and 12 females in vials containing the appropriate experimental medium. On Day 5, the males were discarded, and the female flies were placed on the starvation medium, which consisted of 1% agar in water. Subsequently, the number of dead flies was counted three times a day.

### 2.8 Quantitative RT‐PCR

RNA was isolated from *w* ^*1118*^ females harvested on both Day 4 and 7 using the innuPREP RNA Mini Kit 2.0 (Innuscreen, Berlin, Germany). RNA concentrations were adjusted to 100 ng/μL (Nanodrop, Thermo Fisher Scientific, Dreieich, Germany). RT‐PCR was carried out by using the SensiFAST SYBR No‐ROX One‐step kit (BioCat GmbH, Heidelberg, Germany) and a Rotor‐Gene 6000 RT‐PCR thermocycler system (Qiagen, Düsseldorf, Germany). Primer pairs (Eurofins Genomics, Ebersberg, Germany) and cycling conditions are listed in Table S2. The mRNA levels of the target gene were adjusted to that of the housekeeping gene *RpL32* by 2^−∆∆Ct^ calculation [51].

### 2.9 Statistics

All statistical analyses were conducted using GraphPad Prism software version 10.2.3 (RRID:SCR_002798). Differences between two treatment groups were assessed using unpaired *t*‐tests when data met assumptions of normality and homogeneity of variances, as determined by the Shapiro–Wilk test and *F*‐test, respectively. When either assumption was violated, appropriate alternative tests were employed: the Mann–Whitney test was used for non‐normally distributed data, and the *t*‐test with Welch's correction was applied in cases of unequal variances. For comparisons involving more than two groups, one‐way analysis of variance (ANOVA) was conducted when residuals were normally distributed (assessed by the Shapiro–Wilk test) and variances were homogeneous (assessed by the Brown–Forsythe test). When these assumptions were met, ANOVA was followed by an appropriate multiple comparisons test. In cases of unequal variances, Welch's ANOVA followed by Dunnett's T3 multiple comparisons test was used. If residuals were not normally distributed, the Kruskal–Wallis test was applied, followed by Dunn's multiple comparisons test. To evaluate the effects of two independent variables and their potential interaction, two‐way ANOVA was performed followed by an appropriate multiple comparisons test. Comprehensive details regarding the specific statistical tests used, the number of independent experiments, total number of animals, and the corresponding significance levels are provided in the respective figure and table legends. Results were considered statistically significant at a significance level of *α* = 0.05 (*p < 0.05; **p < 0.01; ***p < 0.001).

## 3 Results

### 3.1 Dietary Spermidine Supplementation Reduces the TAG Level in Female Fruit Flies

**FIGURE 1:**
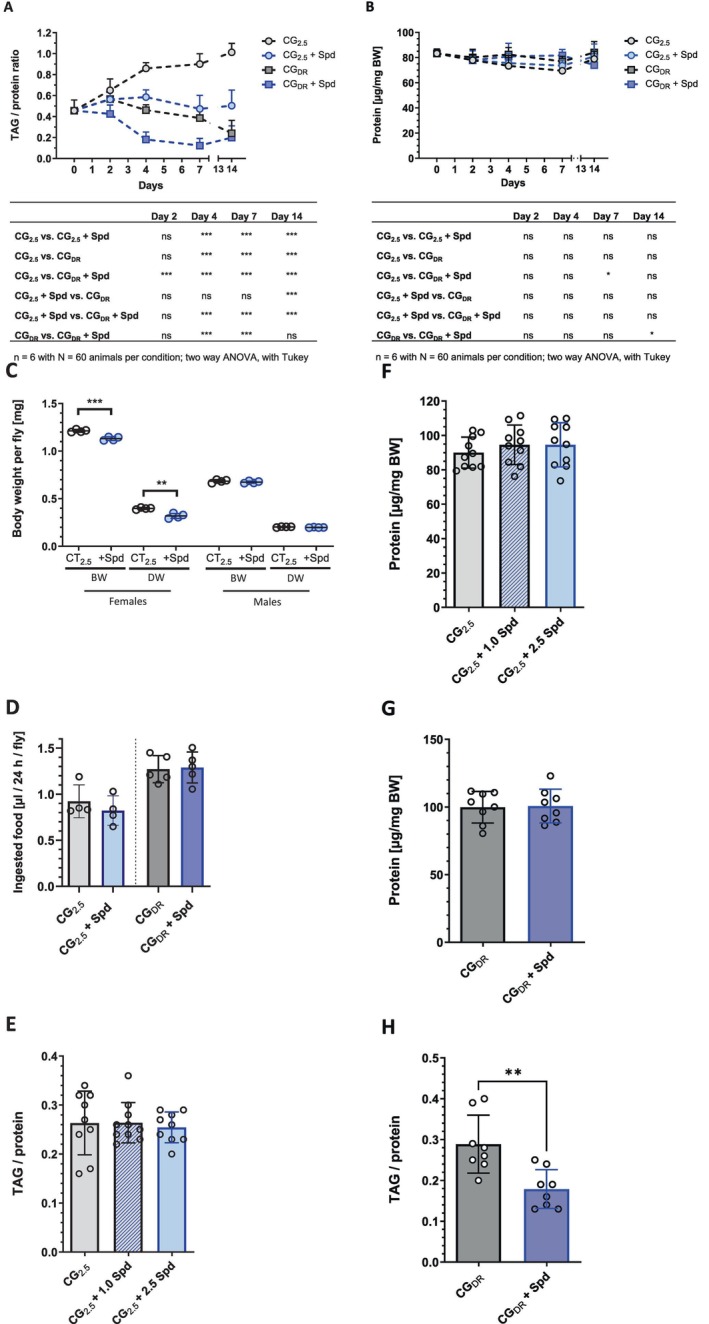
Spermidine supplementation prevents TAG accumulation in adult *D. melanogaster* . (A, B) The TAG, but not the protein content, of female *w* ^*1118*^ fruit flies fed a CG_2.5_ diet showed a gradual increase over the first 14 days post‐eclosion. Both the administration of 2.5 mM spermidine (Spd) and feeding a dietary restriction diet (CG_DR_) inhibited the build‐up of fat storage. Combined, they showed an additive effect leading to a further accelerated reduction in TAG reserves. Freshly eclosed male and female fruit flies were transferred as mixed populations to the specified experimental diets. The animals were harvested at the time points indicated, sorted by sex, and the protein content and the TAG/protein ratio of females were determined at the respective time points. Data points represent the mean of *n* = 6 experiments with *N* = 60 animals per condition and time point. For statistical analyses, two‐way ANOVA with Tukey's multiple comparisons test was carried out. (C) The body weight of females, but not of male fruit flies that were treated with 2.5 mM Spd for a duration of 7 days was decreased. Freshly eclosed male and female fruit flies were transferred as mixed populations to the specified experimental diets, and their fresh (BW) and dry (DW) body weights were determined on Day 7. The experiment was performed in *n* = 4 replicates, with *N* = 400 individuals per condition. Statistical significance was assumed at **p < 0.01, ***p < 0.001 (unpaired *t*‐test). (D) The food intake was not affected by the administration of Spd. Freshly eclosed male and female fruit flies were transferred as mixed populations to the specified experimental diets. On Day 3, their food intake was determined by the Ex‐Q assay described in the “Material and method” section. Data represent the mean ± standard deviation of *n* = 4 independent replications with *N* = 72–84 animals per diet. Statistical analyses were performed by unpaired *t*‐test and Mann–Whitney test, respectively. (E–H) Male *w* ^*1118*^ fruit flies responded to the administration of 2.5 mM Spd with a reduction in their TAG stores only under dietary restriction conditions. The protein and TAG‐to‐protein ratios of males were determined on Day 7 post‐eclosion. The experiment was conducted in *n* = 8–9 replicates with *N* = 80–90 animals per condition. Bars represent the mean ± standard deviation. Statistical significance was assumed at *p < 0.05, ***p < 0.001 (in (E, F) evaluated by one‐way ANOVA followed by Tukey's multiple comparisons test; in (G, H) evaluated by unpaired *t*‐test). CG_2.5_, cornmeal glucose control medium; CG_DR_, dietary restriction medium.

Female *w* ^*1118*^ fruit flies fed on a CG_2.5_ medium post‐eclosion exhibited a gradual accumulation of their TAG reserves over time (Figure 1A). Initially, the TAG level (expressed as a TAG‐to‐protein ratio) was determined to be 0.46 ± 0.10, which progressively increased to a value of 1.01 ± 0.09 by Day 14. In the case of *w* ^*1118*^ females fed on the alternative CT_2.5_ medium, the TAG values displayed a similar trend, starting with 0.69 ± 0.06 post‐eclosion and reaching 1.08 ± 0.12 by Day 7 (Figure S1A). Supplementing either CG_2.5_ or CT_2.5_ medium with 2.5 mM Spd prevented the accumulation of TAG and even led to a time‐dependent reduction of TAG levels in female fruit flies (Figure 1A, Figure S1A). On Day 7, the mean TAG levels of Spd‐treated animals were 0.47 ± 0.13 and 0.43 ± 0.09 compared to controls on CG_2.5_ medium and CT_2.5_ medium with TAG‐to‐protein ratios of 0.90 ± 0.10 and 1.08 ± 0.12, respectively. An extension of the Spd treatment until Day 14 did not lead to further substantial changes in the TAG content of the flies. The TAG‐lowering effect of dietary Spd was confirmed by an additional analytical method, namely via TLC (Figure S1C). To examine whether this is a general effect of dietary Spd on the TAG reserves in fruit flies, we tested females of the *D. melanogaster* wild‐type strain, Oregon RC. Similar to *w* ^*1118*^, we obtained significantly reduced TAG levels by Spd supplementation after 7 days of feeding (Figure S1D). In contrast, the protein levels of female fruit flies were not affected by Spd supplementation (Figure 1B, Figure S1B). As depicted in Figure S1E,F, dietary Spd led to a concentration‐dependent decline of the TAG levels in *w* ^*1118*^ females.

The lower TAG content was accompanied by a slight reduction in both wet and dry body weight of Spd‐treated females, determined on Day 7 (Figure 1C). These analyses additionally revealed that Spd administration did not affect the water content of fruit flies. Moreover, the changes in the TAG levels could not be attributed to food aversion, as the food intake of fruit flies was unchanged upon 2.5 mM Spd supplementation (Figure 1D).

Previous studies reported that the diet provided during larval development can affect the body composition, in particular the fat stores of adult fruit flies [44]. We therefore used different feeding protocols, which either included or did not include a change of medium after the larval stage and a subsequent 7‐day feeding period for adult fruit flies. As shown in Figure S1E,F, a lifelong exposure to CG_2.5_ medium supplemented with 1.0 or 2.5 mM Spd, starting from eggs to 7‐day‐old adult females, did not lead to a more pronounced reduction of the fat stores than a 7‐day treatment with the same Spd concentrations, which started in adult animals only after the eclosion from the pupae. Moreover, switching to the CG_2.5_ control medium after feeding Spd during larval development had no impact on the TAG level of 7‐day‐old females when compared to controls fed the CG_2.5_ diet lifelong (Figure S1E,F). These data indicate that the TAG‐reducing effect of Spd on female *D. melanogaster* therefore only occurs when fed during the adult stage. In good agreement with that, we found that rearing fruit flies from egg to pupa on a 1.0 mM or 2.5 mM Spd‐containing medium did not alter the TAG and protein content nor the body weight of L3 larvae (Figure S1G–I) or change the egg‐to‐pupae developmental time (data not shown). Hence, the TAG‐lowering effect of dietary Spd seems to be specific to female adult flies.

Compared to female fruit flies, age‐matched males fed on control diets exhibited a lower fat content with a TAG‐to‐protein ratio of 0.26 ± 0.07 for CG_2.5_ medium (Figure 1E,F) or 0.52 ± 0.08 for CT_2.5_ medium (Figure S1J,K). In contrast to females, their TAG stores as well as their wet and dry body weights were not significantly affected by Spd supplementation when maintained on these media (Figure 1C,E,F, Figure S1J,K).

### 3.2 Spermidine‐Supplementation and Dietary Restriction Act Independently to Reduce TAG Levels in Adult Female *Drosophila*

Next, we examined the impact of Spd on the body composition of fruit flies fed a diet with a reduced energy content. For this, we used a CG_DR_ diet generated by diluting normal CG_2.5_ food by a factor of two. Female fruit flies kept on this CG_DR_ medium post‐eclosion showed a similar decline in their TAG levels in the first 7 days as those females that were fed a CG_2.5_ diet supplemented with 2.5 mM Spd (Figure 1A). On Day 7, their TAG‐to‐protein value was reduced by about 55% compared to the CG_2.5_ control. Supplementing the CG_DR_ diet with 2.5 mM Spd additionally accelerated the TAG decline in females within the first 7 days of treatment to a TAG‐to‐protein value of 0.12 ± 0.07 (Figure 1A). After a further 7 days of Spd treatment, the TAG content remained almost unchanged. Beginning the feeding with the CG_DR_ or the Spd‐supplemented CG_DR_ diets from larvae onwards led to comparable TAG contents in the 7‐day‐old female flies (Figure S1L,M).

The CG_DR_ diet had previously been employed to study the impact of larval nutrition on adult body composition [44]. Compared to data obtained for the CG_2.5_ diet, feeding the CG_DR_ diet slightly retarded larval development (data not shown) and resulted in migratory L3 larvae with a lower body weight and a reduced protein content (Figure S1G–I), the latter being responsible for the increased TAG‐to‐protein ratio of the CG_DR_ group (Figure S1I). Consistent with the findings in [44], we also observed an increase in the TAG‐to‐protein ratio in adult flies from 0.89 ± 0.15 to 1.13 ± 0.23 (*p* = 0.023, *t*‐test) when larval development took place on CG_DR_ medium, followed by an immediate transition of freshly eclosed adult flies to a standard CG_2.5_ diet for a feeding period of 1 week (Figure S1M). Adding Spd to the CG_DR_ diet did not further affect the egg‐to‐larvae development (data not shown) nor the body composition of L3 larvae (Figure S1G–I). Moreover, exposure to Spd‐supplemented CG_DR_ medium during the larval stages did not influence the body composition of adult females that were transferred to standard CG_2.5_ medium for 7 days post‐eclosion (Figure S1L,M). We conclude that for DR diets with a reduced energy content, Spd must be administered post‐eclosion and not during the larval stages to achieve an additive fat‐lowering effect in adult female flies.

As aforementioned, the TAG depot of male fruit flies did not respond to Spd supplementation under standard CG_2.5_ food conditions. However, they became sensitive to dietary Spd when a CG_DR_ diet was provided, as a 2.5 mM Spd supplementation for 7 days led to a significant reduction in the TAG content (Figure 1G). Again, akin to females, the protein concentration remained unaffected by dietary Spd (Figure 1H), and the administration of Spd during adulthood, rather than during the larval stages, was crucial for the decline in the TAG content (data not shown).

### 3.3 Spermidine‐Induced TAG Reduction Causes Starvation‐Sensitivity in Female Flies

**FIGURE 2:**
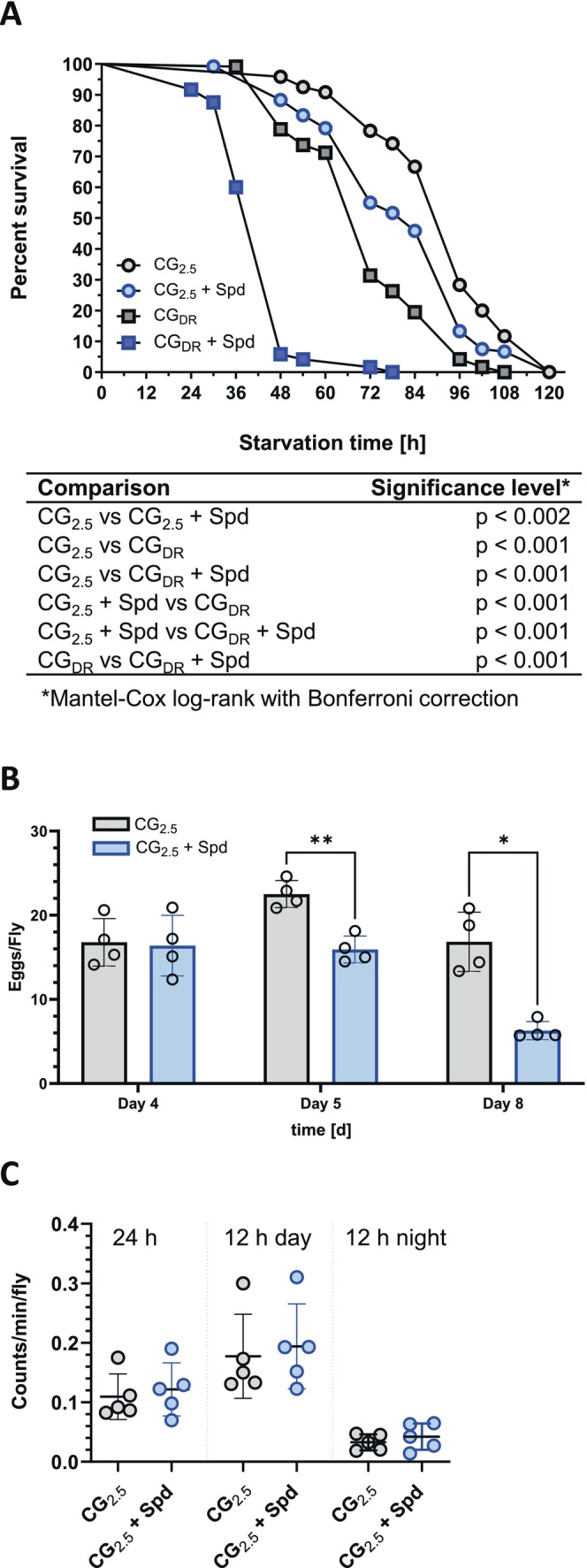
Administration of spermidine leads to enhanced starvation sensitivity and reduced egg laying in *D. melanogaster* females. Freshly eclosed male and female fruit flies were transferred as mixed populations to the specified experimental diets. (A) On Day 7, the animals were sorted by sex, and females in groups of 20 flies per vial were exposed to starvation conditions (1% agar). Their survival was monitored over time. Data points represent the mean of *n* = 4 experiments performed in triplicate with *N* = 240 animals per condition. Survival curves were compared using Log‐rank (Mantel‐Cox) test with Bonferroni correction. (B) To assess the egg laying rate of females, freshly eclosed male and female fruit flies were transferred as mixed populations to the specified experimental diets. For determination of egg laying, two mated females were transferred to new vials with respective experimental diets for 24 h. The eggs laid on Days 4, 5, and 8 were counted. Bars represent the mean standard deviation of four independent experiments with five replicates and *N* = 40 females per condition and time point. Statistical significance was assumed at *p < 0.05, **p < 0.01 (evaluated by two‐way ANOVA followed by Sidak's multiple comparisons test). (C) For the determination of spontaneous locomotor activity, female fruit flies of the different experimental groups were separated from males on Day 4, before they were transferred in groups of 20 flies to the activity monitor vials containing the same diet. The locomotor activity was determined on Day 5. Five independent experiments were conducted in duplicate with 200 individuals per condition (unpaired *t*‐test). CG_2.5_, cornmeal glucose medium with 2.5% inactive yeast extract; CG_DR_, CG medium with a 50% reduced content of cornmeal, glucose, and inactive dry yeast; Spd, spermidine.

The reduced TAG contents of female fruit flies that were fed a Spd‐supplemented CG_2.5_‐diet for a duration of 7 days caused a significantly increased starvation sensitivity when compared to 7‐day‐old CG_2.5_ control flies (Figure 2A). In good accordance, age‐matched flies of the CG_DR_ group, which exhibit a similar reduction in their TAG stores, also displayed a similar response to starvation stress. As flies fed a CG_DR_ diet supplemented with 2.5 mM Spd displayed the lowest TAG content among all treatment groups, they exhibited the fastest mortality kinetics, with more than 90% dead flies already 48 h after being transferred to starvation conditions (Figure 2A). In comparison, the flies that were fed on non‐supplemented CG_DR_ medium survived the stress almost twice as long. Hence, the *ad libitum* fed TAG level of the different treatment groups correlated with their degree of resistance against starvation stress.

### 3.4 The Reduced TAG Content of Spermidine‐Treated Females Is Not Attributable to Increased Egg Production or Locomotor Activity

To elucidate whether Spd‐treated females utilized their TAG stores to a greater extent for energy‐demanding processes, we examined the potential impact of Spd administration on egg production or spontaneous locomotor activity. As shown in Figure 2B, females treated with Spd exhibited a decline in their egg‐laying rates when monitored on Days 4, 5, and 8. Moreover, their locomotor activity on Day 5 was not altered (Figure 2C). Taken together, these findings do not support a model in which the lowered TAG storage in Spd‐treated female fruit flies is caused by up‐regulation of energy‐demanding processes, namely egg production or spontaneous locomotion.

### 3.5 Spermidine Supplementation Counteracts High‐Sugar Diet Mediated Obesity

**FIGURE 3:**
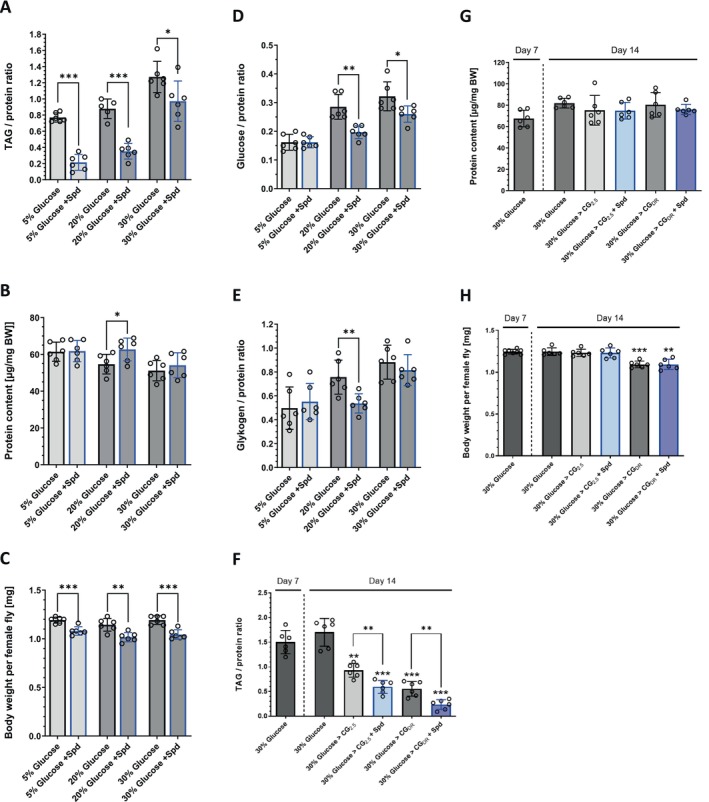
Spermidine supplementation counteracts high‐sugar diet‐induced obesity in *D. melanogaster* females. (A–E) Spermidine (Spd) at 2.5 mM prevented TAG accumulation in female fruit flies when co‐administered with a high‐glucose diet based on standard cornmeal glucose medium containing 2.5% inactive yeast extract (CG_2.5_). Freshly eclosed male and female flies were transferred as mixed populations to the specified experimental diets. On Day 7, the animals were harvested and sorted by sex. The body weight as well as the protein, TAG, glucose, and glycogen content of females were determined. Spd effects were assessed relative to a corresponding control diet with the same sugar content. Data points represent the mean of *n* = 6 experiments with *N* = 60 animals per condition. Statistical significance was assumed at *p < 0.05, **p < 0.01, ***p < 0.001 (evaluated by unpaired *t*‐test or Mann–Whitney test). (F–H) Supplementation of 2.5 mM Spd accelerated the reduction of TAG stores in obese female fruit flies. Freshly eclosed male and female fruit flies were cultured as mixed populations on a 30% high‐glucose diet based on the CG_2.5_ standard diet. After 7 days, one group of flies was harvested, while the rest of the fly populations were transferred to the specified diets and maintained for another 7 days before they were harvested. The harvested flies were sorted by sex before the body weight, protein, and TAG content were determined. The experiment was conducted in *n* = 6 replicates with *N* = 60 animals per condition. Bars represent the mean ± standard deviation. Statistical significance was assumed at *p < 0.05, **p < 0.01, ***p < 0.001 (evaluated by Welch‐ANOVA followed by Dunnett's T3 multiple comparisons test (F) and Kruskal–Wallis test followed by Dunn's multiple comparisons test (G, H)). In (F), the effect of Spd treatment on the TAG‐to‐protein ratio for each medium was additionally evaluated by unpaired *t*‐tests.

Next, we posed the question of whether Spd‐supplementation has the ability to counteract the accumulation of TAG stores in *D. melanogaster* that are fed on an HSD. As shown in Figure 3, Figure S2, an increase in the sugar content of the diet from 5% to 20% and 30%, respectively, led to elevated TAG levels in female fruit flies after 7 days of treatment. This was irrespective of the offered dietary sugar type, namely glucose (Figure 3A), fructose (Figure S2A), or sucrose (Figure S2F), except for the fruit flies fed the 20% fructose diet, which displayed a reduced TAG to protein ratio compared to the 5% fructose group. In all cases, adding 2.5 mM Spd to the different HSDs significantly counteracted TAG accumulation and reduced the body weight of female fruit flies. Remarkably, organismic glucose (Figure 3D, Figure S2D,I) and glycogen levels (Figure 3E, Figure S2E,J) were also diminished in *Drosophila* females by Day 7 when Spd was added to the HSD, while protein levels remained largely unchanged (Figure 3B, Figure S2B,G).

To examine whether Spd also exhibits TAG‐reducing properties in fruit flies that have already manifested an obesity phenotype, freshly eclosed females were initially fed with a 30% high‐glucose diet for a duration of 7 days. This dietary intervention led to flies which displayed a markedly enhanced TAG‐to‐protein ratio of 1.50 ± 0.22 (Figure 3F). When these obese flies were subsequently transferred to a standard GC_2.5_ or CG_DR_ diet for an additional 7 days, their TAG levels were markedly lowered to values of 0.93 ± 0.13 and 0.56 ± 0.14, respectively. Of note, in both media, administration of 2.5 mM Spd significantly further accelerated the decline in TAG contents (Figure 3F). The protein levels remained unaffected by any treatment, while the body weight of the fruit flies decreased when the energy supply was diminished by providing CG_DR_ media (Figure 3G,H). We conclude that Spd supplementation both prevents the accumulation of TAG stores under HSD conditions and promotes the decline of TAG stores in already obese *D. melanogaster* females.

### 3.6 Spermidine Supplementation Reverses Starvation Resistance but Not the Reduced Egg Production in Fruit Flies Fed a High‐Sugar Diet

**FIGURE 4:**
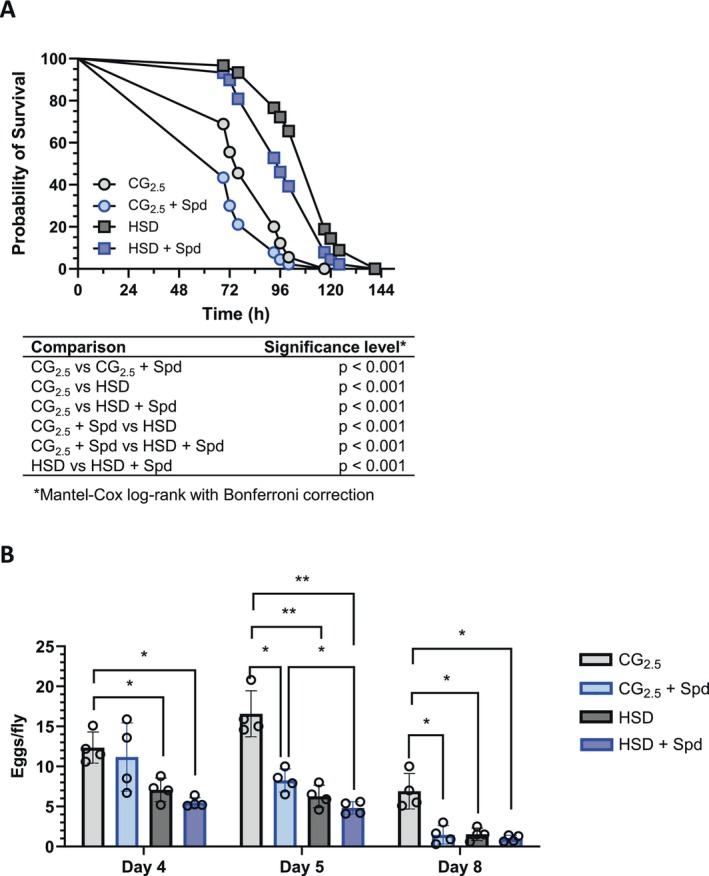
Administration of spermidine causes increased starvation sensitivity but does not impact the reduced egg‐laying rate of *D. melanogaster* females fed a high‐sugar diet. Freshly eclosed male and female fruit flies were transferred as mixed populations to the specified experimental diets. (A) On Day 7, the animals were sorted by sex, and females in groups of 20 flies per vial were exposed to starvation conditions (1% agar). Their survival was monitored over time. Data points represent the mean of *n* = 4 experiments performed in triplicate with *N* = 240 animals per condition. Survival curves were compared using the Log‐rank (Mantel‐Cox) test with Bonferroni correction. (B) To assess the egg‐laying rate of females, freshly eclosed male and female fruit flies were transferred as mixed populations to the specified experimental diets. For the determination of egg laying, two mated females were transferred to new vials with respective experimental diets for 24 h. The eggs laid on Days 4, 5, and 8 were counted. Bars represent the mean standard deviation of four independent experiments with five replicates and *N* = 40 females per condition and time point. Statistical significance was assumed at *p < 0.05, **p < 0.01 (evaluated by two‐way ANOVA followed by Tukey's multiple comparisons test). CG_2.5_: Cornmeal glucose medium with 2.5% inactive yeast extract; HSD, high‐sugar diet; Spd, spermidine.

The increased TAG levels determined for HSD‐fed fruit flies were found to be correlated with an enhanced resistance to starvation (Figure 4A). Consistent with the observed fat‐lowering activity, the supplementation of HSD with Spd led to an increased sensitivity of female fruit flies towards starvation (Figure 4A). In contrast, the adverse effect of an HSD on egg production was not reversed by Spd supplementation (Figure 4B). These data suggest that the actual TAG level modulated by dietary Spd determines the resilience against starvation, but not the egg production rate of adult female fruit flies.

### 3.7 Adipokinetic Hormone Signaling Is Required for Both Dietary Restriction‐ and Spermidine‐Induced TAG Modulation

**FIGURE 5:**
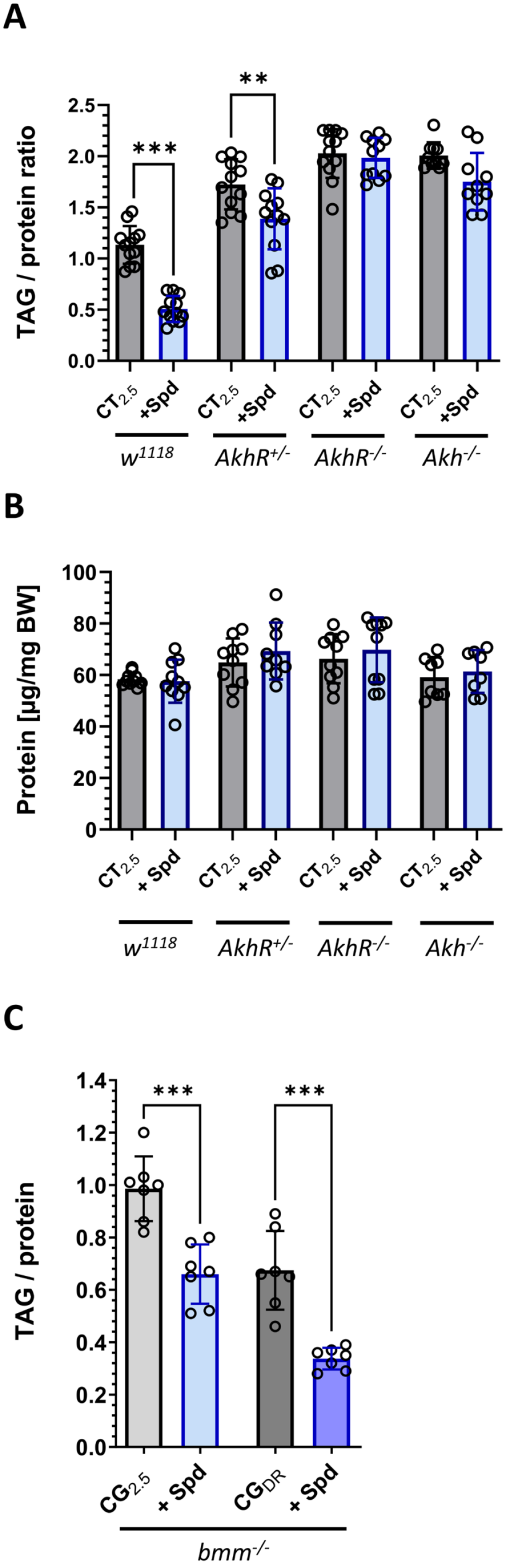
The TAG‐reducing effect of spermidine depends on a functional Akh pathway but not on Bmm. Freshly eclosed male and female fruit flies of the indicated genotypes were transferred as mixed populations to the specified experimental diets. On Day 7, the animals were harvested and sorted by sex before the protein and TAG content of females were determined. (A, B) Bars represent the mean of *n* = 8–10 experiments with *N* = 80–100 animals per condition. (C) Bars represent the mean of *n* = 7 experiments with *N* = 42–49 animals per condition. Statistical analyses were performed using an unpaired *t*‐test to determine the impact of spermidine (Spd) supplementation on the body composition for each genotype and dietary condition (**p < 0.01, ***p < 0.001). *Akh*, *adipokinetic hormone*; *AkhR*, *adipokinetic hormone receptor*; *bmm*, *brummer TAG lipase*.

The catabolic adipokinetic hormone (Akh) plays a crucial role in the regulation of the energy stores in the fruit fly [9]. Hence, we next examined whether the Spd‐dependent decline of TAG levels in female fruit flies is mediated by the Akh pathway. As illustrated in Figure 5A,B, the TAG level of both *Akh* ^*−/−*^ and *AkhR* ^*−/−*^ mutants, which lack the Akh hormone and its receptor, respectively, is unresponsive to Spd administration in a CT_2.5_. This indicates that Akh signaling is necessary to reduce the TAG content through dietary Spd. However, when Spd was added under DR conditions, a slight but significant decline in the TAG‐protein‐ratio was observed in *AkhR* ^*−/−*^ mutants (Figure S3). Moreover, we tested whether the TAG lipase Brummer (Bmm), which acts in parallel to Akh/AkhR, is involved in this process. However, feeding the *bmm* ^*−/−*^ null mutant with a CG_2.5_ medium containing 2.5 mM Spd still had a significant TAG‐reducing effect (Figure 5C). In summary, we conclude that the Akh/Akhr pathway, but not Bmm, is required to elicit the TAG‐reducing effect of dietary Spd in adult *D. melanogaster* .

### 3.8 The Transcript Level of the Acetyl‐CoA Carboxylase Gene (*Acc*) Involved in the Formation of Fatty Acid Is Up‐Regulated by a 7‐Day Treatment With Spermidine

**FIGURE 6:**
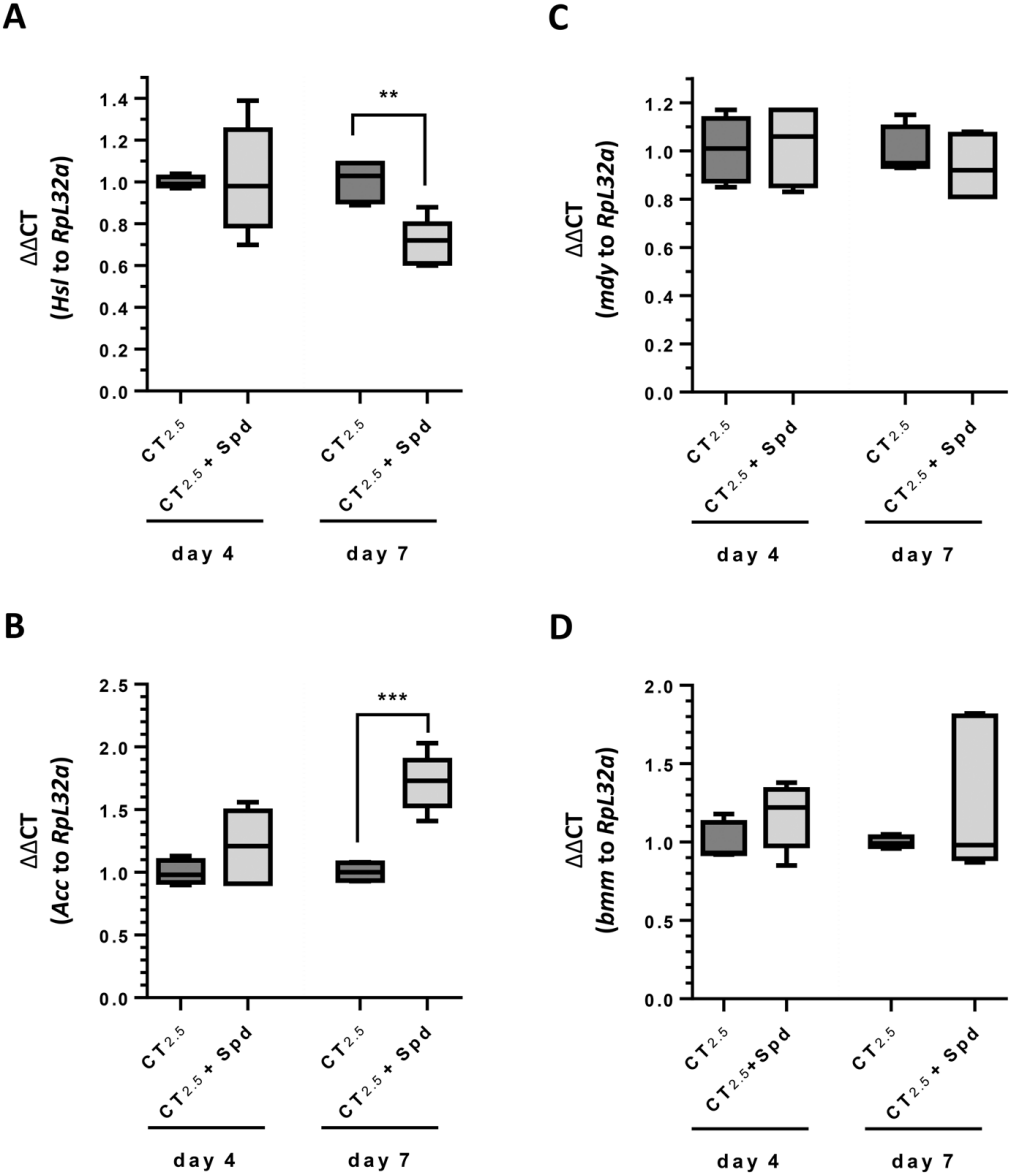
The transcript levels of *D. melanogaster* genes involved in the formation of fatty acid and TAG are elevated by spermidine administration on Day 7. Freshly eclosed *D. melanogaster* *w* ^*1118*^ females were randomly allocated in groups of 10 animals to Caltech medium containing 2.5% inactive yeast extract (CT_2.5_) or to the same medium supplemented with 2.5 mM spermidine (Spd). After 4 and 7 days, the flies were harvested. Following RNA isolation, quantitative RT‐PCR analyses were carried out for the indicated genes and the housekeeping gene *RpL32*. Relative expression levels were calculated by the ΔΔCT method. Box plot bars represent (*n* = 6). Statistical significance was assessed by *t*‐test (B–D) and Welch's *t*‐test (A) (**p < 0.01). *Acc*, *acetyl‐CoA carboxylase*; *B* *mm*, *brummer TAG lipas*e; *Hsl*, *hormone‐sensitive lipase*; *mdy*, *midway*, diacylglycerol O‐acyltransferase; *RpL32*, *ribosomal protein L32*.

Given that *Akh* and *AkhR* null mutants did not respond with a decrease in their fat stores to Spd administration, we proceeded to examine whether the expression of key factors involved in TAG metabolism was influenced by Spd treatment. To this end, we determined the transcript levels of the anabolic genes *Acc* (fatty acid synthesis) and *mdy* (TAG synthesis) as well as of the catabolic genes *Hsl* (sterol ester breakdown) and *bmm* (TAG breakdown) in *D. melanogaster* females on both Day 4 and 7 (Figure 6A–D). The Spd treatment over a period of 4 days did not significantly alter the expression levels of these transcripts when compared to CG_2.5_ controls. However, by Day 7, the fatty acid synthesis gene *Acc* was significantly up‐regulated by 1.7‐fold, whereas the transcript level of the sterol ester hydrolase *Hsl* was downregulated to a value of 0.71. The transcript levels of *mdy* and *bmm* remained unaffected. Hence, we conclude that the lack of TAG accumulation observed within the first week post‐eclosion cannot be explained by altered expression levels of these key genes of TAG homeostasis.

### 3.9 The Proportion of Yeast Extract in the Diet Is Crucial for the Fat‐Lowering Effect of Spermidine

**FIGURE 7:**
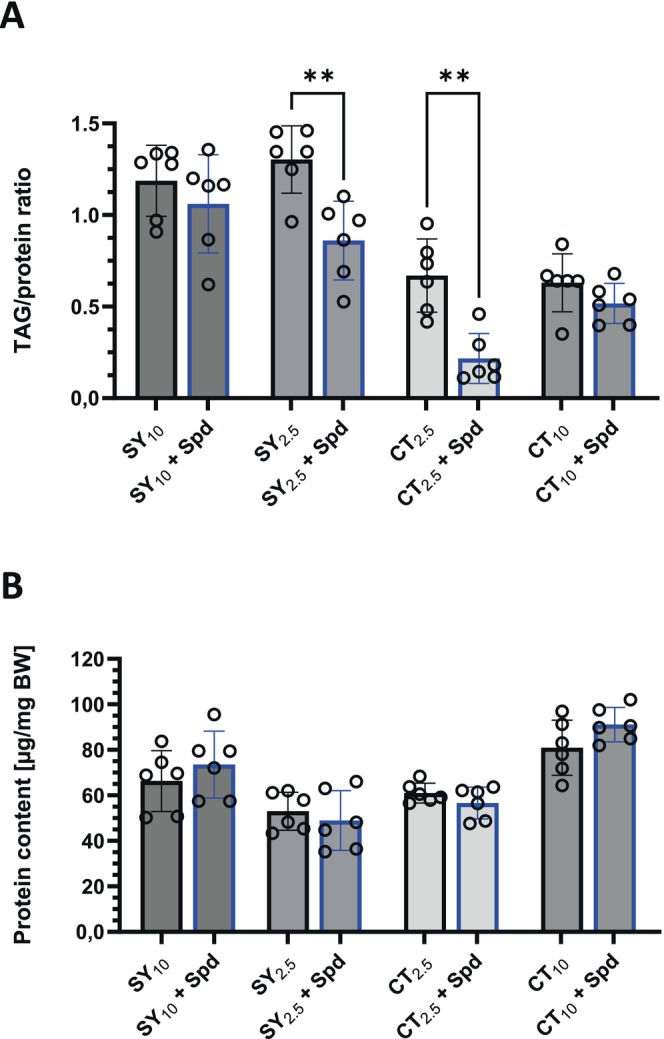
Increasing the proportion of inactive yeast extract in the *Drosophila* diet abolishes the TAG‐lowering effect of spermidine. Freshly eclosed male and female fruit flies were transferred as mixed populations to the specified experimental diets. On Day 7, the animals were harvested and sorted by sex before the protein and TAG content of females were determined. (A) In both diets, Caltech (CT) and sucrose‐yeast (SY), a content of 2.5% inactive yeast extract promoted the spermidine (Spd)‐dependent decline of TAG stores, while a proportion of 10% inactive yeast extract abolished the Spd effect. (B) The protein content remained unaffected by dietary Spd. Bars represent the mean ± SD of *n* = 3 experiments in duplicate with *N* = 60 animals per condition. Statistical analyses were performed using an unpaired *t*‐test to determine the effects of Spd supplementation on the body composition parameters for the different experimental diets.

Both the CT_2.5_ and GC_2.5_ media that have been utilized in our Spd‐supplementation studies up to this point contain a 2.5% proportion of inactive yeast extract. The SY_10_ medium, another commonly employed laboratory diet for *Drosophila*, is distinguished by an inactive yeast extract content of 10%. When we added 2.5 mM Spd to this diet, the TAG‐reducing effect of Spd on female fruit flies was completely abolished (Figure 7A). Likewise, the protein content remained again unaffected following Spd administration (Figure 7B). Hence, we then decided to modify the inactive yeast extract proportion in both SY and CT media. Elevating the inactive yeast extract content of the CT diet to 10% resulted in the TAG content remaining unchanged under a Spd treatment for 7 days (Figure 7A). On the other hand, when the inactive yeast extract proportion in the SY medium was reduced to 2.5%, administration of Spd for 7 days led to significantly lowered TAG levels in female fruit flies (Figure 7A). Together, these data suggest that a lower inactive yeast extract content in the diet favors TAG reduction through supplemented Spd in adult female fruit flies.

### 3.10 Spermidine Supplementation of the Low‐Yeast CT_2.5_ Diet Does Not Elevate Endogenous Spermidine Level of *w* ^*1118*^ Females but Promotes the Formation of Catabolic Polyamines

Next, we asked which consequences dietary Spd supplementation has on the polyamine profile of *w* ^*1118*^ fruit flies. As shown in Table 1, 7‐day‐old *Drosophila* females fed on a CT_2.5_ diet contained Spd as the predominant polyamine. Moreover, smaller amounts of putrescine, N^1^‐acetyl‐Spd, and spermine were detectable. Remarkably, when the CT_2.5_ diet was supplemented with 2.5 mM Spd, the endogenous Spd content of the flies was not affected with 0.833 ± 0.145 nmol/mg BW versus 0.802 ± 0.100 nmol/mg BW for the control and 2.5 mM Spd‐treated group, respectively. However, the supplemented Spd elicited drastic changes in the pattern of the other di‐ and polyamines. The putrescine and N^1^‐acetyl‐Spd levels were found to be increased by about 7‐fold and 3‐fold, respectively, while the spermine concentration was slightly diminished. N‐acetylputrescine and other catabolic metabolites such as N^8^‐acetylspermidine or N^1^‐acetylspermine were not detectable in *D. melanogaster* under any of the dietary conditions.

**TABLE 1:** The dietary content of inactive yeast and the administration of spermidine affects the polyamine pattern of female *D. melanogaster* .

Polyamines [nmol/mg BW]				
Diet	Put	N1AcSpd	Spd	Spm
CT_2.5_	0.045 ± 0.007	0.030 ± 0.003	0.833 ± 0.145	0.093 ± 0.014
CT_2.5_ +Spd	0.328 ± 0.056***	0.098 ± 0.028***	0.802 ± 0.100^ns^	0.068 ± 0.003***
Fold change	7.29	3.27	0.96	0.73
CT_10_	0.046 ± 0.008	0.022 ± 0.004	1.960 ± 0.252	0.139 ± 0.022
CT_10_ +Spd	0.131 ± 0.033***	0.053 ± 0.006***	2.291 ± 0.161*	0.161 ± 0.087^ns^
Fold change	2.85	2.41	1.17	1.16

*Note:* Freshly eclosed fruit flies were allocated in groups of 12 males and 12 females to the indicated experimental diets for 7 days before they were harvested. The polyamine profiles of females were determined by HPLC analysis, and for each polyamine, the fold‐change induced by dietary Spd was calculated. Data represent the mean ± SD of *n* = 8–9 experiments with *N* = 96–108 individuals per condition. Statistical significance was assumed at *p < 0.05, ***p < 0.001 (evaluated using unpaired *t*‐test, if necessary, with Welch's correction or Mann–Whitney test).

Abbreviations: N1AcSpd, N^1^‐acetyl spermidine; ns, not significant; Put, putrescine; Spd, spermidine; Spm, spermine.

### 3.11 Spermidine Supplementation of the High‐Yeast CT_10_ Diet Elevates Endogenous Spermidine Level of *w* ^*1118*^ Females With Reduced Formation of Catabolic Polyamines

Compared to female fruit flies maintained on a CT_2.5_ medium, 7‐day‐old females fed the CT_10_ diet with a higher inactive yeast extract content showed more than 2‐fold higher Spd levels of 1960 ± 0.252 nmol/mg BW, while the concentrations of putrescine and N^1^‐acetyl‐Spd were found to be almost similar. Unlike the data for the CT_2.5_ diet, the addition of 2.5 mM Spd to a CT_10_ diet resulted in a significantly further elevation of the endogenous Spd content (Table 1). Moreover, the increase in the levels of other polyamines was less pronounced than in those females fed the Spd‐supplemented CT_2.5_ diet, namely 3‐fold for putrescine and less than 2.5‐fold for N^1^‐acetyl‐Spd. In conclusion, our data suggest that the amount of inactive yeast extract present in the diet is a key factor that determines how supplemented Spd alters the polyamine profile of *D. melanogaster* .

### 3.12 Spermidine Supplementation Does Not Elevate the Transcript Levels of Putative *Sat* and *pao* Genes in *D. melanogaster*

**FIGURE 8:**
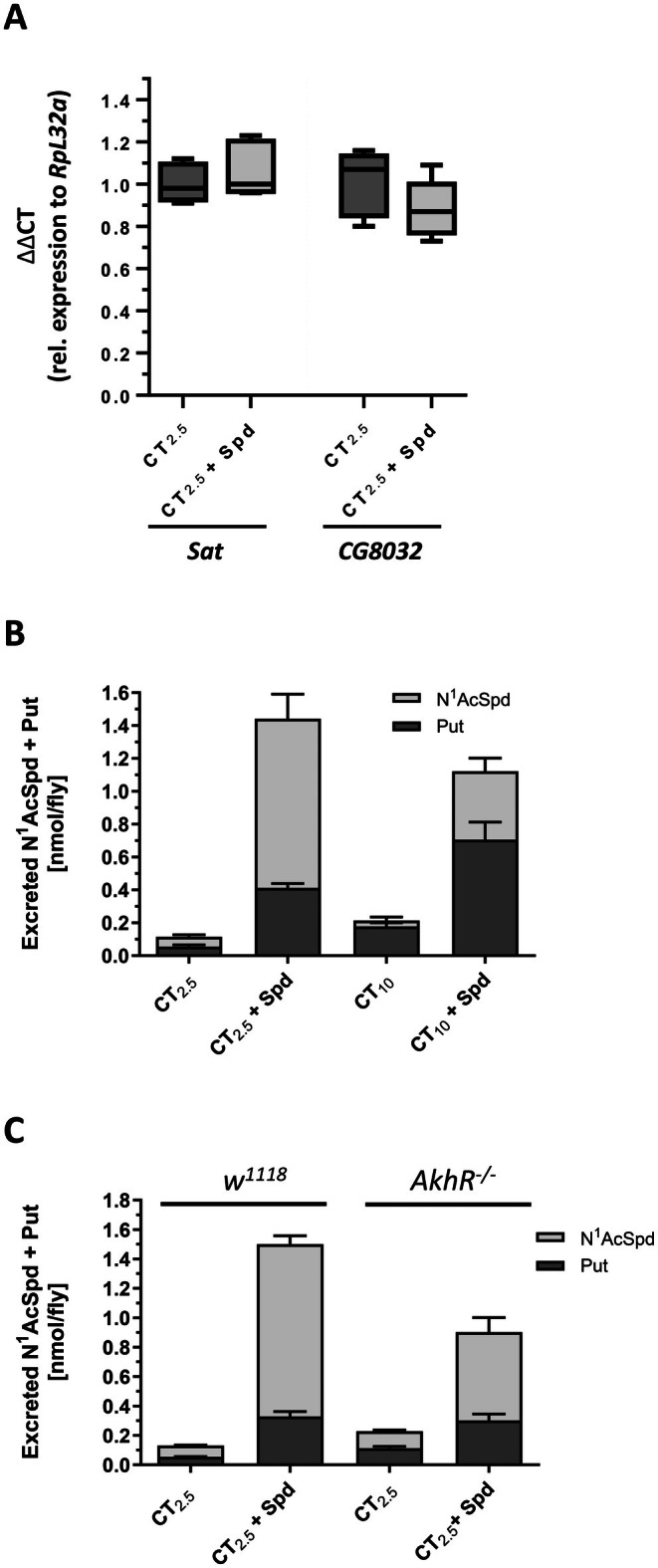
The amount of the spermidine degradation products putrescine and N^1^‐acetylspermidine excreted by female *D. melanogaster* depends on dietary factors and the genotype. (A) The transcript level of the putative SSAT encoding gene *Sat* and the putative *pao* gene *CG8032* from *D. melanogaster* were not affected by spermidine (Spd) administration. Freshly eclosed *D. melanogaster* *w* ^*1118*^ females were allocated in groups of 10 animals to CT_2.5_ medium or to the same medium supplemented with 2.5 mM Spd. After 4 days, the flies were harvested. Following RNA isolation, quantitative RT‐PCR analyses were carried out for the two indicated genes and the housekeeping gene *RpL32*. Relative expression levels were calculated by the ΔΔCT method. Unpaired *t*‐test (*n* = 5). (B, C) Freshly eclosed fruit flies of the indicated genotypes were allocated in groups of 12 males and 12 females to the different experimental diets for 3 days, before the females were transferred for 24 h to Ex‐Q vials containing the corresponding experimental diets. On day 4, the flies were removed, before their excreta were rinsed off the tube walls. The polyamine profiles of the excreta were determined by HPLC analysis. The bars represent the mean ± SD of three independent experiments with *N* = 30 individuals. The statistical analyses (two‐way ANOVA followed by Tukey's multiple comparisons test) are shown in Tables S3 and S4. *AkhR*, *Adipokinetic hormone receptor*; CT_2.5_, Caltech medium containing 2.5% inactive yeast extract; CT_10_, Caltech medium containing 10% inactive yeast extract; PAO, polyamine oxidase; Put, putrescine; *RrpL32*, *Ribosomal protein L32*; SSAT, spermidine/spermine N^1^‐acetyltransferase; N1AcSpd, N^1^‐acetylspermidine.

In mammals, the enzymes SSAT (encoded by the *sat1* gene) and PAO are responsible for the N^1^‐acetylation of Spd and the further interconversion to putrescine. Transcription of the mammalian *sat1* is strongly induced by polyamines, while *pao* expression occurs constitutively and is less regulated [27]. It was recently suggested that the corresponding *D. melanogaster* homologues are encoded by the genes CG4210, named *Sat*, and CG8032 [41]. However, our qRT‐PCR analyses revealed that the transcript levels of both genes were not affected in response to a 7‐day administration of 2.5 mM Spd compared to the CG_2.5_ controls (Figure 8A), although significantly increased levels of N^1^‐acetyl‐Spd and putrescine were determined in these treated fruit flies. It therefore remains unclear whether these genes are involved in Spd degradation.

### 3.13 The Excretion Rate of Putrescine and N^1^ ‐Acetylspermidine Depends on the Yeast Content of the Fruit Fly Diet

The elevated levels of putrescine and N^1^‐acetyl‐Spd found in Spd‐supplemented female fruit flies are most probably products of the Spd catabolism, which is presumably activated in these flies to counteract an excessive increase in endogenous Spd levels. In mammals, a surplus of polyamines can be counter‐regulated through increased cellular export and subsequent renal excretion of N‐acetylated di‐ and polyamines [52]. Hence, we proceeded to investigate whether there was evidence for a similar adaptation in *D. melanogaster* . To this end, we analyzed the excretion patterns of polyamines from fruit flies. As depicted in Figure 8B, females consuming a CT_2.5_ diet excreted only low amounts of putrescine (0.054 ± 0.007 nmol/fly/24 h) and N^1^‐acetyl‐Spd (0.062 ± 0.013 nmol/fly/24 h), whereas administration of 2.5 mM Spd led to a drastic increase of both compounds in the excreta, namely a 7.5‐fold and 17.5‐fold elevation for putrescine and N^1^‐acetyl‐Spd, respectively.

Interestingly, when compared to female flies fed the CT_2.5_ control diet, the feces of female fruit flies consuming a CT_10_ diet contained about three times more putrescine and a comparable amount of N^1^‐acetyl‐Spd (Figure 8B). It was found that the addition of 2.5 mM Spd to the CT_10_ diet increased putrescine excretion of females with a value of 0.673 nmol/fly/24 h to a similar level as in Spd‐supplemented CT_2.5_ fed females. However, the amount of excreted N^1^‐acetyl‐Spd was only half that of females fed a 2.5 mM Spd‐supplemented CT_2.5_ diet (Figure 8B).

Transfer experiments depicted in Figure S4 show that a large proportion of the Spd ingested with the food is expelled by *Drosophila* immediately via the gut and that the fruit fly does not excrete endogenous Spd in relevant quantities under any of the examined dietary conditions. Likewise, spermine or N‐acetylated di‐ and polyamines (N‐acetylputrescine, N^8^‐acetylspermidine, or N^1^‐acetylspermine) were not detectable in the excreta of any experimental group. Collectively, these results indicate that Spd supplementation results in a higher formation and excretion rate of N^1^‐acetyl‐Spd, particularly when the proportion of the inactive yeast extract of the diet is low.

### 3.14 Spermidine Supplementation Elevates the Endogenous Spermidine Pool in *AkhR* Null Mutants While Leading to Lower N^1^ ‐Acetylspermidine Excretion

Female *AkhR* ^*−/−*^ flies that consumed the CG_2.5_ diet for 7 days contained about 25% higher Spd levels than *w* ^*1118*^ counterparts (*p* = 0.016, evaluated by *t*‐test with Welch's correction; Table 2), whereas the putrescine, N^1^‐acetyl‐Spd, and spermine concentrations were comparable between the two fly strains. Moreover, we found that when the CG_2.5_ diet was supplemented with 2.5 mM Spd, the endogenous Spd pool of the *AkhR* ^*−/−*^ mutants was further elevated by 30%. This is in contrast to the unchanged Spd levels in *w* ^*1118*^ flies that were fed a Spd‐supplemented CG_2.5_ diet, but is reminiscent of the situation observed in *w* ^*1118*^ flies that had consumed a Spd‐supplemented CG_10_ diet (see above). Of note, the elevated Spd level of the *AkhR* ^*−/−*^ flies was accompanied by smaller changes in the putrescine, N^1^‐acetyl‐Spd, and spermine levels when compared to the corresponding *w* ^*1118*^ flies (Table 2). In good accordance, the Spd‐treated *AkhR* ^*−/−*^ flies excreted less N^1^‐acetyl‐Spd (Figure 8C).

**TABLE 2:** Changes of the polyamine profile in response to spermidine administration in *Adipokinetic hormone receptor* (*AkhR* ^*−/−*^) mutants.

Polyamines [nmol/mg BW]				
Strain/Diet	Put	N1AcSpd	Spd	Spm
*w* ^*1118*^/CG_2.5_	0.065 ± 0.018	0.034 ± 0.010	1.187 ± 0.095	0.129 ± 0.020
*w* ^*1118*^/CG_2.5_ +Spd	0.474 ± 0.081***	0.168 ± 0.062***	1.120 ± 0.155^ns^	0.082 ± 0.013***
Fold change	6.87	4.88	0.94	0.64
*AkhR* ^*−/−*^/CG_2.5_	0.083 ± 0.024	0.045 ± 0.010	1.493 ± 0.247	0.143 ± 0.021
*AkhR* ^*−/−*^/CG_2.5_ +Spd	0.161 ± 0.051***	0.108 ± 0.034***	1.814 ± 0.280*	0.132 ± 0.023^ns^
Fold change	2.01	2.39	1.21	0.96

*Note:* Females of *w* ^*1118*^ and *AkhR* ^*−/−*^ mutant were fed for 7 days a CG_2.5_ control diet or a CG_2.5_ diet that was supplemented with 2.5 mM Spd. Polyamines were determined by HPLC analyses. Data represent the mean ± SD of *n* = 7–8 experiments, each with *N* = 70–80 animals. Statistical significance was assumed at *p < 0.05; ***p < 0.001 (evaluated using an unpaired *t*‐test, if necessary, with Welch's correction).

Abbreviations: N1AcSpd, N^1^‐acetylspermidine; ns, not significant; Put, putrescine; Spd, spermidine; Spm, spermine.

## 4 Discussion

Here, we report on the TAG‐reducing activity of dietary Spd in *D. melanogaster* , where we observed stage‐, sex‐, and diet‐specific effects. Regarding stage specificity, it was found that administering Spd exclusively in adulthood was necessary and sufficient to elicit significantly decreased TAG levels in females, whereas the fat depots during larval growth remained unaffected by Spd supplementation. We suggest that this phenomenon is due to differences in energy metabolism between larvae and adults in *Drosophila* [7]. The larvae engage in continuous feeding to support their rapid growth and, at the same time, build up an energy reservoir, including TAG, which is stored in the larval fat body for mobilization during subsequent periods without food intake [53]. This anabolic status is illustrated by the fact that the loss of the catabolic Akh pathway, which leads to an increased TAG level in adult fruit flies, was reported to not affect the fat stores of the larvae [15]. During the metamorphosis process, the larval fat body disassembles into single adipocyte cells, some of which are still present for a short time in the haemolymph of newly eclosed adults [53, 54]. Hence, post‐eclosion, adult flies must build up a new TAG storage in the emerging adult fat body whose adipocytes originate from a different cell line than those of the larval fat body [53]. In our standard protocol for Spd feeding studies with adult flies, we started experiments with freshly eclosed individuals, which are characterized by two different fat depots: remnants of the larval TAG reserve and the developing adult TAG store. Based on data from previous reports [53, 54], we can exclude that the Spd‐triggered reduction of TAG stores is related to residual larval fat depots, since larval fat cells were found to be largely eliminated in both sexes within the first 24 h after eclosion. The TAG‐reducing effect of 2.5 mM Spd on female flies, however, became evident only by Day 4 of treatment. In adult flies, the primary role of the adult fat body is to sustain organismal energy homeostasis, which is achieved, inter alia, through greater hormonal regulation by Akh signaling [7], thereby possibly enabling the lipid‐lowering effect of Spd (see below).

Our data revealed a distinct degree of sex‐specificity regarding the fat‐lowering effect of dietary Spd in *D. melanogaster* . Male fruit flies, which stored less TAG than females, were found to be less sensitive towards Spd, as they responded to the administered Spd only on DR diet with a reduced carbohydrate and protein content. Remarkably, unlike in females, the DR diet alone did not lead to a reduction of TAG storage in males either. However, male fruit flies were previously found to mobilize their TAG stores more rapidly than females under starvation conditions [54]. Differences in food intake rates between the sexes may be a factor influencing the differential effect of Spd on the TAG stores in female and male fruit flies. Mated female flies consume significantly more food than males [11]. To date, very little is known about sex‐specific effects of Spd administration on fat accumulation in mammals. This is because the vast majority of mouse studies investigating this subject were conducted exclusively on male animals [31, 32, 33, 34, 35, 36, 37]. In a mouse study that includes both sexes and in which Spd was administered intraperitoneally on a daily basis, thereby avoiding differences in food intake, similar fat‐reducing effects were observed in both males and females [55]. However, a sex‐specific effect was still observed, as Spd treatment improved insulin sensitivity only in male but not in female mice. Another related sex‐specific effect was reported by [30], where SSAT overexpression reduced total fat mass in both sexes, but an increase in fat‐free body mass was observed exclusively in females. Similar to the male–female difference in fat storage found in mammals [56], a distinct sexual dimorphism concerning TAG homeostasis is manifested in *D. melanogaster* [54]. Many genes involved in whole‐body TAG homeostasis were found to be differentially expressed. In particular, the TAG lipase Bmm was shown to be a major contributor to the sexual dimorphism in TAG depots that develop between the sexes in *D. melanogaster* post‐eclosion [54]. When maintained under standard culture conditions, a constant decline in TAG levels within the first week after eclosion—similar to the Spd effect on female TAG stores—was only observed in male flies, resulting in considerably lower TAG levels compared to age‐matched females. Concurrently, the male flies showed an increased expression of *bmm* compared to the females, which was considered one of the main underlying factors for the difference in body composition between the sexes. Hence, we suggest that the catabolic status of TAG metabolism in males during early adulthood may limit the potential of dietary Spd to achieve the same degree of TAG reduction observed in females. Of note, in Spd‐treated male mice, an increased expression of the orthologous *ATGL* gene was observed in visceral fat [55]. However, although these data make Bmm a possible candidate, the TAG lipase is most likely not a key driver in Spd‐induced TAG degradation in females, as following Spd administration, *bmm* transcript levels in *w* ^*1118*^ flies remained unaffected, and female *bmm* loss‐of‐function mutants responded with reduced TAG levels. Accordingly, *bmm* deficiency was previously reported to have little to no effect on TAG levels and TAG mobilization in females [54].

Supplemented Spd not only prevented the onset of obesity under HSD (30% sugar) but also facilitated the reduction of TAG stores in already obese female fruit flies. These findings align well with recent mouse studies, which demonstrated that the addition of Spd to the diet led to improved TAG levels, especially in obesity models. Schipke et al. [33] fed mice a high‐sugar diet containing 35% sucrose. The obesity induced by this diet was efficiently mitigated by a combination of voluntary physical activity and the administration of 3 mM Spd to their drinking water. In four additional feeding studies that investigated the anti‐obesity potential of Spd [35, 36, 37, 55], mice were fed high‐fat diets (HFD) containing 42%, 45%, 32% and 34.9% fat, respectively. Treatment with Spd prevented excessive weight gain of HFD‐mice, mainly due to a reduced formation of fat stores without affecting food intake. Given that Spd was administered intraperitoneally in all cases through daily injections of 20 or 50 mg/kg doses over a period of 1, 3, or 6 months, an involvement of the intestinal microbiota can be ruled out. Ma et al. [32] examined the influence of dietary Spd on mice that had become already obese due to a 16‐week pre‐feeding period on an HFD. Similar to our findings in fruit flies, they observed a reduction in body weight in Spd‐treated animals, which was administered at a dose of 20 mg/kg via the drinking water for an additional 8 weeks. The decrease in weight gain among Spd‐treated mice was largely attributed to a significant loss of fat stores, while muscle mass was even slightly increased. Similarly, we found that the protein levels of fruit flies were not affected by Spd administration across all experimental conditions and at any time point examined.

In accordance with previous studies [7, 9], our data confirm the correlation between the current TAG level of the flies and their ability to withstand starvation. This relationship also applied to HSD flies with elevated TAG levels, as they showed increased survival rates during starvation. This increased resilience of HSD flies was attenuated by the administration of Spd in the HSD, which concurrently lowered TAG levels. Interestingly, such a relation was not evident in terms of egg laying. It has been established that obesity negatively affects female fertility across various animal species [57]. Accordingly, we observed that feeding female fruit flies with an HSD resulted in reduced egg production. Nevertheless, female fertility could not be restored by the administration of Spd in the HSD, even though the TAG levels in these flies were significantly reduced. This finding aligns with a recent study that investigated egg laying in several genetic obesity models of the fruit fly, using different dietary regimens [57]. The authors concluded that elevated TAG levels per se were not the cause of reduced female fertility in *Drosophila*. Instead, increased sugar levels in response to HSD were suggested to lead to impairment of egg production. Of note, in our HSD‐fed females that received Spd, not only the TAG level but also the glycogen and glucose levels were reduced. Hence, these findings suggest an alternative mechanism independent of internal TAG and glucose status by which HSD negatively affects female fertility.

We found that the TAG‐lowering activity of Spd depends on the actual yeast extract content of the diet. Remarkably, the latter value also correlated with the endogenous Spd level of fruit flies. Compared to females maintained on the low‐yeast diet containing 2.5% yeast extract, feeding the corresponding high‐yeast diet with a concentration of 10% of the yeast extract led to a more than 2‐fold enhanced Spd level in female fruit flies. The yeast extract is particularly important for the supply of protein, although it also contributes other essential and non‐essential biofactors, including various lipids and micronutrients such as vitamins and minerals [42, 43]. Therefore, when present at a concentration of 10%, the yeast extract provides sufficient nutrients to the fly to increase metabolic processes, and since polyamines are required in many fundamental processes such as transcription and translation, this includes Spd synthesis. Consistently, a diet rich in yeast extract has been frequently demonstrated to promote an increase in fecundity in *D. melanogaster* [46, 58]. Remarkably, a recent study reported that supplementing a *Drosophila* diet that contained 12.5% yeast extract with 5 mM Spd led to an enhanced organismal Spd level and a concomitant elevated egg‐laying rate [46]. In accordance, we found that when additional dietary Spd was administered under high‐yeast feeding conditions, the endogenous Spd level was even enhanced, while the TAG level remained unaffected. Of note, the observed slight increase of catabolic polyamines N^1^‐acetyl‐Spd N1AcSpd indicates that counter‐regulation has already been initiated in response to Spd administration (see below).

When fruit flies were fed a low‐yeast diet containing 2.5% yeast extract, the lower dietary nutrient supply was reflected in a lower endogenous Spd level, again indicating that polyamine synthesis was adapted to the current nutritional status. Nevertheless, the TAG stores of the flies were constantly built up over time under these dietary conditions. However, we found that supplementing the low‐yeast diet with Spd triggered catabolic processes. These catabolic processes include homeostatic regulation of the Spd status, which is seen by the drastically enhanced levels of putrescine and N^1^‐acetyl‐Spd in the fly, particularly in their excreta.

In contrast to the well‐studied mammalian SSAT and PAO, which are responsible for the degradation of excess Spd, the respective fruit fly genes have not yet been functionally identified. Nevertheless, we determined the impact of Spd administration to the putative *Drosophila* homologues of SSAT and PAO, which are encoded by the genes *Sat* and *CG8032*, respectively, and whose transcription levels have been recently assessed in polyamine synthesis mutants of the fruit fly [41]. The transcript levels of both genes were not affected by Spd supplementation, which speaks against them being involved in polyamine catabolism. In line with this, *Sat* encodes an N‐acetyltransferase that is more closely related to the previously characterized thialysine N^ɛ^‐acetyltransferases (also known as SSAT2), for which neither Spd nor spermine is a physiological substrate [59, 60]. The presence of the conserved amino acid residues Ser(81), Thr(82), and Leu(129) in the *Drosophila* protein that distinguish thialysine N^ɛ^‐acetyltransferases from SSAT1 are particularly important here [59]. Moreover, the work on these closely related N‐acetyltransferases has also revealed that, during evolution, Spd‐preferring SSAT1 generally first developed in vertebrates, which suggests that N^1^‐acetyl‐Spd formation in flies is not governed by Sat but by an unrelated enzyme. We want to note at this point that, despite the qRT‐PCR results, we cannot rule out the involvement of the potential PAO with respect to the observed Spd interconversion, especially since PAO is not the regulatory enzyme in mammalian polyamine catabolism and its regulation can also take place post‐transcriptionally. In mammals, the catabolic arm of polyamine metabolism plays a crucial role in the pathologies of several diseases and may also serve as a potential therapeutic target [27]. It will therefore be exciting to expand the knowledge about the genes and proteins involved in polyamine degradation in the *Drosophila* model, particularly the identification of the enzyme(s) responsible for the N^1^‐acetylation of Spd.

Female flies fed a low‐yeast diet containing 2.5% yeast extract responded to the administration of dietary Spd with increased production and excretion of catabolic polyamine metabolites, but their endogenous Spd content was not altered despite the high supply of Spd in the food. More intriguingly, there was no TAG accumulation over time, and the TAG store even declined with Spd administration. Hence, the addition of dietary Spd to the low‐yeast diet led to a mismatch between the nutritional status and the Spd present, which triggered catabolic processes also beyond polyamine degradation. Accordingly, when Spd was supplemented to a DR diet with reduced carbohydrate and protein content (compared to a 2.5% yeast extract diet), which alone already caused a TAG reduction in females, the TAG stores of fruit flies were further reduced, demonstrating an additive effect. This effect is also reflected in the reduced survival under starvation.

The observed relationship between homeostatic regulation of the endogenous Spd pool and the metabolism of TAG stores shows certain parallels to findings obtained in mice that overexpress SSAT [28, 29, 30]. In these animals, an increased N^1^‐acetylation and further degradation/excretion of Spd were also associated with a depletion of TAG stores. Intriguingly, there is compensatory polyamine synthesis pathway up‐regulation in response to SSAT overexpression, which results in a constant endogenous Spd level in this transgenic mouse model. In sum, this led to an accelerated futile turnover in the cyclic polyamine metabolism at the expense of energy consumption. On the one hand, large quantities of polyamine precursors, for example, SAM, were therefore required; on the other hand, there was a significant increase in the consumption of acetyl‐CoA due to the constantly high SSAT activity [28, 29, 30]. As a result of lowered acetyl‐CoA levels, fatty acid synthesis was impaired, and the transgenic mice exhibited a lean phenotype reflected by reduced white adipose tissue mass. Remarkably, a knock‐down of nicotinamide N‐methyltransferase in white adipose tissue and liver of mice resulted in a similar increase in futile polyamine flux, accompanied by elevated urinary excretion of acetylated polyamines, which also has an anti‐obesity effect [61]. Our data indicate that the acetyl‐CoA consumption in *D. melanogaster* was likewise enhanced by providing dietary Spd. However, in our set‐up, it is unlikely that a futile polyamine cycle process was initiated, as the endogenous Spd pool was replenished by dietary source. Further studies are therefore required to elucidate whether a diminished acetyl‐CoA pool caused the lean phenotype observed in Spd‐treated fruit flies.

Our assumption that a disproportionate relationship between the current Spd level and the nutrient status triggers catabolic processes is further supported by our data on *Akh*/*AkhR* loss‐of‐function mutants fed a low‐yeast diet. The glucagon‐like peptide hormone Akh functions as a key catabolic hormone regulating body fat content and haemolymph sugar levels, hence representing a major lipolysis system in *D. melanogaster* [9]. Akh is released into the haemolymph under negative energy balance conditions or if energy‐demanding processes need to be initiated and maintained [9]. Changes in the TAG content in response to Akh secretion are mediated via the cognate G protein‐coupled receptor AkhR expressed on the fat cells and the corresponding downstream effectors, including one or more currently uncharacterized TAG lipase(s), which execute TAG lipolysis. In line with previous studies [15, 18], both female *Akh* and *AkhR* null mutants contained increased TAG stores. Of note, as shown here, they also have a significantly elevated Spd level when compared to corresponding *w* ^*1118*^ females. Moreover, Spd supplementation of the low‐yeast diet did not affect the TAG level and even increased the endogenous Spd pool in the *AkhR* null mutants. Therefore, Akh/AkhR signaling is required for the Spd‐dependent decrease of TAG stores in fruit flies and is probably also involved in the regulation of Spd catabolism. Our analyses revealed no changes in transcript levels of selected candidate genes involved in lipid and fatty acid metabolism that could account for the TAG‐reducing effect of Spd; therefore, it remains unclear how the Akh pathway mediates TAG regulation upon Spd treatment. The observed up‐regulation of the fatty acid synthesis gene *Acc* and downregulation of the sterol ester lipase *Hsl* in *w* ^*1118*^ females by day 7 of the Spd treatment are possibly secondary effects of the low TAG level. *Acc* up‐regulation may explain the slightly enhanced TAG level found in Spd‐treated females by day 14.

A promising candidate pathway, which may mediate the TAG‐lowering effect of Spd and should be tested in future *Drosophila* studies, is the catabolic autophagy pathway. Both dietary Spd and the nutrient status are known regulators of autophagy [62]. In particular, in mice that were fed a hypercaloric diet, the administration of Spd has been reported to reduce both weight gain and obesity‐associated alterations via induction of autophagy [35]. A similar beneficial effect on body weight and body composition in high‐fat diet mice was observed in another study [55], where Spd was given by daily injection. However, by testing Spd in different genetic backgrounds, it was concluded that its metabolic effects are most likely not mediated by a single pathway but rather arise through comprehensive changes that engage multiple pathways.

In this *Drosophila* study, we discovered a link between the yeast extract concentration, the main protein source of the diet, and the fat‐reducing activity of Spd administration. Very recently, the interaction between dietary protein intake and Spd supplementation in the fruit fly was investigated in the context of aging [46]. Low dietary protein content or Spd supplementation extended the life expectancy of female *w* ^*1118*^ flies, and both also counteracted the mitochondrial aging process in the brain. Still, it was assumed that the underlying mechanisms are very likely distinct, as indicated by the presence of additive effects observed in experiments that combined both nutritional regimes. In the present study, we did not assess lifespan or senescence‐related parameters. Our findings therefore relate specifically to metabolic phenotypes and fat storage, without direct conclusions about aging. The concentration of 2.5 mM Spd applied in the current study falls within a similar range to previous studies in invertebrate models, including *D. melanogaster* [45, 46], and in mammals [31, 33, 45], where beneficial impacts on health and lifespan were observed. It must be regarded as a pharmacological concentration that cannot be reached by diet. It will be tempting to elucidate whether the observed relationship can be confirmed in mammalian obesity models. If the efficacy of Spd against obesity can also be modulated by protein supply in these models, then this might have implications for the treatment of obesity in humans.

## Author Contributions

Kai Lüersen, Thomas Röder, and Gerald Rimbach contributed to the study conception and design. Material preparation, data collection, and analysis were performed by Celina Runke, Bernhard Blank‐Landeshammer, Julian Weghuber, and Ronald P. Kühnlein. The first draft of the manuscript was written by Kai Lüersen and Celina Runke, and all authors contributed to revised versions of the manuscript. All authors read and approved the final manuscript.

## Conflicts of Interest

The authors declare no conflicts of interest.

## Supporting information


**Figure S1:** fsb271153‐sup‐0001‐FigureS1.pdf.

**Table S1:** fsb271153‐sup‐0002‐TableS1.pdf.

## Data Availability

The data that support the findings of this study are available in the Materials and Methods, Results, and/or Supporting Information S1 of this article.

## References

[1] Y. Choksomngam , S. Pattanakuhar , N. Chattipakorn , and S. C. Chattipakorn , “The Metabolic Role of Spermidine in Obesity: Evidence From Cells to Community,” Obesity Research & Clinical Practice 15 (2021): 315–326, 10.1016/j.orcp.2021.06.009. 34217652

[2] L. Xu , Q. Yang , and J. Zhou , “Mechanisms of Abnormal Lipid Metabolism in the Pathogenesis of Disease,” International Journal of Molecular Sciences 25 (2024): 8465, 10.3390/ijms25158465. 39126035 PMC11312913

[3] WHO , “Obesity and Overweight,” 2025.

[4] K. P. Lee , S. J. Simpson , F. J. Clissold , et al., “Lifespan and Reproduction in *Drosophila*: New Insights From Nutritional Geometry,” Proceedings of the National Academy of Sciences of the United States of America 105 (2008): 2498–2503, 10.1073/pnas.0710787105. 18268352 PMC2268165

[5] S. Staats , K. Lüersen , A. E. Wagner , and G. Rimbach , “ *Drosophila melanogaster* as a Versatile Model Organism in Food and Nutrition Research,” Journal of Agricultural and Food Chemistry 66 (2018): 3737–3753, 10.1021/acs.jafc.7b05900. 29619822

[6] V. Eickelberg , K. Lüersen , S. Staats , and G. Rimbach , “Phenotyping of *Drosophila melanogaster* –A Nutritional Perspective,” Biomolecules 12 (2022): 221, 10.3390/biom12020221. 35204721 PMC8961528

[7] N. Chatterjee and N. Perrimon , “What Fuels the Fly: Energy Metabolism in *Drosophila* and Its Application to the Study of Obesity and Diabetes,” Science Advances 7 (2021): eabg4336, 10.1126/sciadv.abg4336. 34108216 PMC8189582

[8] M. Galikova and P. Klepsatel , “Obesity and Aging in the *Drosophila* Model,” International Journal of Molecular Sciences 19 (2018): 1896, 10.3390/ijms19071896. 29954158 PMC6073435

[9] C. Heier and R. P. Kühnlein , “Triacylglycerol Metabolism in *Drosophila melanogaster* ,” Genetics 210 (2018): 1163–1184, 10.1534/genetics.118.301583. 30523167 PMC6283168

[10] W. Palm , J. L. Sampaio , M. Brankatschk , et al., “Lipoproteins in *Drosophila melanogaster*–Assembly, Function, and Influence on Tissue Lipid Composition,” PLoS Genetics 8 (2012): e1002828, 10.1371/journal.pgen.1002828. 22844248 PMC3406001

[11] M. H. Sieber and A. C. Spradling , “Steroid Signaling Establishes a Female Metabolic State and Regulates SREBP to Control Oocyte Lipid Accumulation,” Current Biology: CB 25 (2015): 993–1004, 10.1016/j.cub.2015.02.019. 25802149 PMC6894397

[12] M. Carvalho , J. L. Sampaio , W. Palm , M. Brankatschk , S. Eaton , and A. Shevchenko , “Effects of Diet and Development on the *Drosophila* Lipidome,” Molecular Systems Biology 8 (2012): 600, 10.1038/msb.2012.29. 22864382 PMC3421444

[13] J. M. Tennessen , N. M. Bertagnolli , J. Evans , M. H. Sieber , J. Cox , and C. S. Thummel , “Coordinated Metabolic Transitions During *Drosophila* Embryogenesis and the Onset of Aerobic Glycolysis,” G3: Genes, Genomes, Genetics 4 (2014): 839–850, 10.1534/g3.114.010652. 24622332 PMC4025483

[14] M. Galikova and P. Klepsatel , “Endocrine Control of Glycogen and Triacylglycerol Breakdown in the Fly Model,” Seminars in Cell & Developmental Biology 138 (2023): 104–116, 10.1016/j.semcdb.2022.03.034. 35393234

[15] M. Galikova , M. Diesner , P. Klepsatel , et al., “Energy Homeostasis Control in Drosophila Adipokinetic Hormone Mutants,” Genetics 201 (2015): 665–683, 10.1534/genetics.115.178897. 26275422 PMC4596676

[16] M. V. Vatashchuk , M. M. Bayliak , V. V. Hurza , K. B. Storey , and V. I. Lushchak , “Metabolic Syndrome: Lessons From Rodent and Drosophila Models,” BioMed Research International 2022 (2022): 5850507, 10.1155/2022/5850507. 35782067 PMC9242782

[17] M. Lehmann , “Endocrine and Physiological Regulation of Neutral Fat Storage in *Drosophila* ,” Molecular and Cellular Endocrinology 461 (2018): 165–177, 10.1016/j.mce.2017.09.008. 28893568 PMC5756521

[18] S. Grönke , G. Müller , J. Hirsch , et al., “Dual Lipolytic Control of Body Fat Storage and Mobilization in *Drosophil*a,” PLoS Biology 5 (2007): e137, 10.1371/journal.pbio.0050137. 17488184 PMC1865564

[19] S. Grönke , A. Mildner , S. Fellert , et al., “Brummer Lipase Is an Evolutionary Conserved Fat Storage Regulator in *Drosophila* ,” Cell Metabolism 1 (2005): 323–330, 10.1016/j.cmet.2005.04.003. 16054079

[20] K. Igarashi and K. Kashiwagi , “The Functional Role of Polyamines in Eukaryotic Cells,” International Journal of Biochemistry & Cell Biology 107 (2019): 104–115, 10.1016/j.biocel.2018.12.012. 30578954

[21] A. E. Pegg , “Functions of Polyamines in Mammals,” Journal of Biological Chemistry 291 (2016): 14904–14912, 10.1074/jbc.R116.731661. 27268251 PMC4946908

[22] D. H. Bae , D. J. R. Lane , P. J. Jansson , and D. R. Richardson , “The Old and New Biochemistry of Polyamines,” Biochimica et Biophysica Acta, General Subjects 1862 (2018): 2053–2068, 10.1016/j.bbagen.2018.06.004. 29890242

[23] F. Madeo , S. J. Hofer , T. Pendl , et al., “Nutritional Aspects of Spermidine,” Annual Review of Nutrition 40 (2020): 135–159, 10.1146/annurev-nutr-120419-015419. 32634331

[24] B. Ramos‐Molina , M. I. Queipo‐Ortuno , A. Lambertos , F. J. Tinahones , and R. Penafiel , “Dietary and Gut Microbiota Polyamines in Obesity‐ and Age‐Related Diseases,” Frontiers in Nutrition 6 (2019): 24, 10.3389/fnut.2019.00024. 30923709 PMC6426781

[25] N. C. Munoz‐Esparza , M. L. Latorre‐Moratalla , O. Comas‐Baste , N. Toro‐Funes , M. T. Veciana‐Nogues , and M. C. Vidal‐Carou , “Polyamines in Food,” Frontiers in Nutrition 6 (2019): 108, 10.3389/fnut.2019.00108. 31355206 PMC6637774

[26] T. Esatbeyoglu , A. Ehmer , D. Chaize , and G. Rimbach , “Quantitative Determination of Spermidine in 50 German Cheese Samples on a Core‐Shell Column by High‐Performance Liquid Chromatography With a Photodiode Array Detector Using a Fully Validated Method,” Journal of Agricultural and Food Chemistry 64 (2016): 2105–2111, 10.1021/acs.jafc.6b00078. 26915410

[27] R. A. Casero and A. E. Pegg , “Polyamine Catabolism and Disease,” Biochemical Journal 421 (2009): 323–338, 10.1042/BJ20090598. 19589128 PMC2756025

[28] J. Jell , S. Merali , M. L. Hensen , et al., “Genetically Altered Expression of Spermidine/Spermine N^1^‐Acetyltransferase Affects Fat Metabolism in Mice via Acetyl‐CoA,” Journal of Biological Chemistry 282 (2007): 8404–8413, 10.1074/jbc.M610265200. 17189273

[29] C. Liu , O. Perez‐Leal , C. Barrero , et al., “Modulation of Polyamine Metabolic Flux in Adipose Tissue Alters the Accumulation of Body Fat by Affecting Glucose Homeostasis,” Amino Acids 46 (2014): 701–715, 10.1007/s00726-013-1548-3. 23881108 PMC5184767

[30] E. Pirinen , T. Kuulasmaa , M. Pietila , et al., “Enhanced Polyamine Catabolism Alters Homeostatic Control of White Adipose Tissue Mass, Energy Expenditure, and Glucose Metabolism,” Molecular and Cellular Biology 27 (2007): 4953–4967, 10.1128/MCB.02034-06. 17485446 PMC1951486

[31] S. Pankoke , C. Pfarrer , S. Glage , C. Mühlfeld , and J. Schipke , “Oral Supplementation With the Polyamine Spermidine Affects Hepatic but Not Pulmonary Lipid Metabolism in Lean but Not Obese Mice,” Nutrients 14 (2022): 4318, 10.3390/nu14204318. 36297003 PMC9611404

[32] L. Ma , Y. Ni , L. Hu , et al., “Spermidine Ameliorates High‐Fat Diet‐Induced Hepatic Steatosis and Adipose Tissue Inflammation in Preexisting Obese Mice,” Life Sciences 265 (2021): 118739, 10.1016/j.lfs.2020.118739. 33186567

[33] J. Schipke , M. Vital , A. Schnapper‐Isl , D. H. Pieper , and C. Mühlfeld , “Spermidine and Voluntary Activity Exert Differential Effects on Sucrose‐ Compared With Fat‐Induced Systemic Changes in Male Mice,” Journal of Nutrition 149 (2019): 451–462, 10.1093/jn/nxy272. 30715385

[34] Y. Ni , L. Zheng , L. Zhang , et al., “Spermidine Activates Adipose Tissue Thermogenesis Through Autophagy and Fibroblast Growth Factor 21,” Journal of Nutritional Biochemistry 125 (2024): 109569, 10.1016/j.jnutbio.2024.109569. 38185346

[35] A. F. Fernandez , C. Barcena , G. G. Martinez‐Garcia , et al., “Autophagy Couteracts Weight Gain, Lipotoxicity and Pancreatic Beta‐Cell Death Upon Hypercaloric Pro‐Diabetic Regimens,” Cell Death & Disease 8 (2017): e2970, 10.1038/cddis.2017.373. 28771229 PMC5596561

[36] M. Gao , W. Zhao , C. Li , et al., “Spermidine Ameliorates Non‐Alcoholic Fatty Liver Disease Through Regulating Lipid Metabolism via AMPK,” Biochemical and Biophysical Research Communications 505 (2018): 93–98, 10.1016/j.bbrc.2018.09.078. 30241944

[37] D. Wang , J. Yin , Z. Zhou , et al., “Oral Spermidine Targets Brown Fat and Skeletal Muscle to Mitigate Diet‐Induced Obesity and Metabolic Disorders,” Molecular Nutrition & Food Research 65 (2021): e2100315, 10.1002/mnfr.202100315. 34363644

[38] L. Ma , Y. Ni , Z. Wang , et al., “Spermidine Improves Gut Barrier Integrity and Gut Microbiota Function in Diet‐Induced Obese Mice,” Gut Microbes 12 (2020): 1–19, 10.1080/19490976.2020.1832857. PMC7668533 33151120

[39] K. E. Leon , A. M. Fruin , S. L. Nowotarski , and J. R. DiAngelo , “The Regulation of Triglyceride Storage by Ornithine Decarboxylase (Odc1) in Drosophila,” Biochemical and Biophysical Research Communications 523 (2020): 429–433, 10.1016/j.bbrc.2019.12.078. 31870547

[40] T. S. Morales , E. C. Avis , E. K. Paskowski , H. Shabar , S. L. Nowotarski , and J. R. DiAngelo , “The Role of Spermidine Synthase (SpdS) and Spermine Synthase (Sms) in Regulating Triglyceride Storage in Drosophila,” Medical Science 9 (2021): 27, 10.3390/medsci9020027. PMC8162547 34063217

[41] T. S. C. Morales , R. B. Hepp , J. Foley , et al., “Characterizing the Homeostatic Regulation of the Polyamine Pathway Using the *Drosophila melanogaster* Model System,” Gene Reports 24 (2021): 101269.

[42] D. N. A. Lesperance and N. A. Broderick , “Meta‐Analysis of Diets Used inDrosophilaMicrobiome Research and Introduction of theDrosophilaDietary Composition Calculator (DDCC),” G3: Genes, Genomes, Genetics 10 (2020): 2207–2211, 10.1534/g3.120.401235. 32371452 PMC7341119

[43] K. Lüersen , T. Roder , and G. Rimbach , “ *Drosophila melanogaster* in Nutrition Research‐The Importance of Standardizing Experimental Diets,” Genes & Nutrition 14 (2019): 3, 10.1186/s12263-019-0627-9. 30766617 PMC6359822

[44] N. Rehman and J. Varghese , “Larval Nutrition Influences Adult Fat Stores and Starvation Resistance in *Drosophila* ,” PLoS One 16 (2021): e0247175, 10.1371/journal.pone.0247175. 33606785 PMC7895371

[45] T. Eisenberg , H. Knauer , A. Schauer , et al., “Induction of Autophagy by Spermidine Promotes Longevity,” Nature Cell Biology 11 (2009): 1305–1314, 10.1038/ncb1975. 19801973

[46] Y. Liang , A. Krivograd , S. J. Hofer , et al., “Spermidine Supplementation and Protein Restriction Protect From Organismal and Brain Aging Independently,” Aging (Albany NY) 17 (2025): 1429–1451, 10.18632/aging.206267. 40489973 PMC12245200

[47] Q. Wu , G. Yu , S. J. Park , Y. Gao , W. W. Ja , and M. Yang , “Excreta Quantification (EX‐Q) for Longitudinal Measurements of Food Intake in *Drosophila* ,” Iscience 23 (2020): 100776, 10.1016/j.isci.2019.100776. 31901635 PMC6941854

[48] B. Al‐Anzi , V. Sapin , C. Waters , K. Zinn , R. J. Wyman , and S. Benzer , “Obesity‐Blocking Neurons in *Drosophila* ,” Neuron 63 (2009): 329–341, 10.1016/j.neuron.2009.07.021. 19679073 PMC2742587

[49] P. M. Kabra , H. K. Lee , W. P. Lubich , and L. J. Marton , “Solid‐Phase Extraction and Determination of Dansyl Derivatives of Unconjugated and Acetylated Polyamines by Reversed‐Phase Liquid Chromatography: Improved Separation Systems for Polyamines in Cerebrospinal Fluid, Urine and Tissue,” Journal of Chromatography 380 (1986): 19–32, 10.1016/s0378-4347(00)83621-x. 3745383

[50] A. Heinick , K. Urban , S. Roth , et al., “ *Caenorhabditis elegans* P_5B_‐Type ATPase CATP‐5 Operates in Polyamine Transport and Is Crucial for Norspermidine‐Mediated Suppression of RNA Interference,” FASEB Journal 24 (2010): 206–217, 10.1096/fj.09-135889. 19762559 PMC2797033

[51] K. Jans , K. Lüersen , J. von Frieling , T. Roeder , and G. Rimbach , “Dietary Lithium Stimulates Female Fecundity in *Drosophila melanogaster* ,” BioFactors 50 (2024): 326–346, 10.1002/biof.2007. 37706424

[52] A. J. Vargas , E. L. Ashbeck , C. A. Thomson , E. W. Gerner , and P. A. Thompson , “Dietary Polyamine Intake and Polyamines Measured in Urine,” Nutrition and Cancer 66 (2014): 1144–1153, 10.1080/01635581.2014.949801. 25204413

[53] J. R. Aguila , J. Suszko , A. G. Gibbs , and D. K. Hoshizaki , “The Role of Larval Fat Cells in Adult *Drosophila melanogaster* ,” Journal of Experimental Biology 210 (2007): 956–963, 10.1242/jeb.001586. 17337708

[54] L. W. Wat , C. Chao , R. Bartlett , et al., “A Role for Triglyceride Lipase Brummer in the Regulation of Sex Differences in *Drosophila* Fat Storage and Breakdown,” PLoS Biology 18 (2020): e3000595, 10.1371/journal.pbio.3000595. 31961851 PMC6994176

[55] C. Y. Liao , O. M. P. Kummert , A. M. Bair , et al., “The Autophagy Inducer Spermidine Protects Against Metabolic Dysfunction During Overnutrition,” Journals of Gerontology. Series A, Biological Sciences and Medical Sciences 76 (2021): 1714–1725, 10.1093/gerona/glab145. 34060628 PMC8436989

[56] J. C. Link and K. Reue , “Genetic Basis for Sex Differences in Obesity and Lipid Metabolism,” Annual Review of Nutrition 37 (2017): 225–245, 10.1146/annurev-nutr-071816-064827. PMC5759759 28628359

[57] R. D. Nunes and D. Drummond‐Barbosa , “A High‐Sugar Diet, but Not Obesity, Reduces Female Fertility in *Drosophila melanogaster* ,” Development 150 (2023): dev201769, 10.1242/dev.201769. 37795747 PMC10617608

[58] C. Liu , N. Tian , P. Chang , and W. Zhang , “Mating Reconciles Fitness and Fecundity by Switching Diet Preference in Flies,” Nature Communications 15 (2024): 9912, 10.1038/s41467-024-54369-w. PMC11568147 39548088

[59] K. Luersen , “Leishmania Major Thialysine N^E^‐Acetyltransferase: Identification of Amino Acid Residues Crucial for Substrate Binding,” FEBS Letters 579 (2005): 5347–5352, 10.1016/j.febslet.2005.08.063. 16194533

[60] C. S. Coleman , B. A. Stanley , A. D. Jones , and A. E. Pegg , “Spermidine/Spermine‐N1‐Acetyltransferase‐2 (SSAT2) Acetylates Thialysine and Is Not Involved in Polyamine Metabolism,” Biochemical Journal 384 (2004): 139–148, 10.1042/BJ20040790. 15283699 PMC1134097

[61] D. Kraus , Q. Yang , D. Kong , et al., “Nicotinamide N‐Methyltransferase Knockdown Protects Against Diet‐Induced Obesity,” Nature 508 (2014): 258–262, 10.1038/nature13198. 24717514 PMC4107212

[62] S. J. Hofer , A. K. Simon , M. Bergmann , T. Eisenberg , G. Kroemer , and F. Madeo , “Mechanisms of Spermidine‐Induced Autophagy and Geroprotection,” Nature Aging 2 (2022): 1112–1129, 10.1038/s43587-022-00322-9. 37118547

