# Supplementary material for: Homeostatic Counter‐Regulation Mediates Spermidine‐Induced Triacylglyceride Reduction in Drosophila melanogaster—From Phenotype to Molecular Mechanism
Source: FASEB J. 2025 Oct 21;39(20):e71153. doi: 10.1096/fj.202502620R (PMC12539389; doi:10.1096/fj.202502620R)
Supplement: Supplementary file 1 — Figure S1: fsb271153‐sup‐0001‐FigureS1.pdf. [file FSB2-39-e71153-s001.pdf]

Supplemented Figures

Fig. S1A

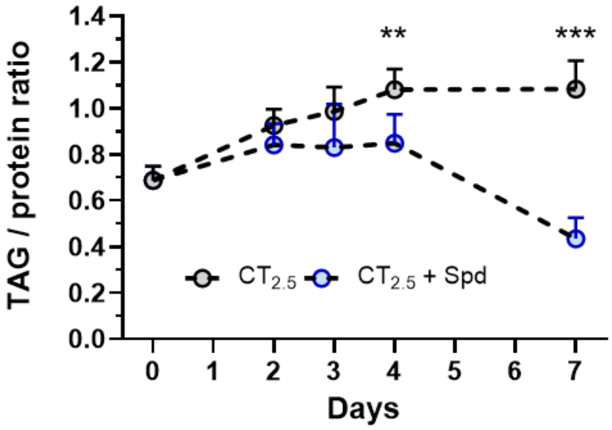

$CT_{2.5}$ vs $CT_{2.5}$ + Spd	
Day 2	ns
Day 3	ns
Day 4	**
Day 7	****

Fig. S1B

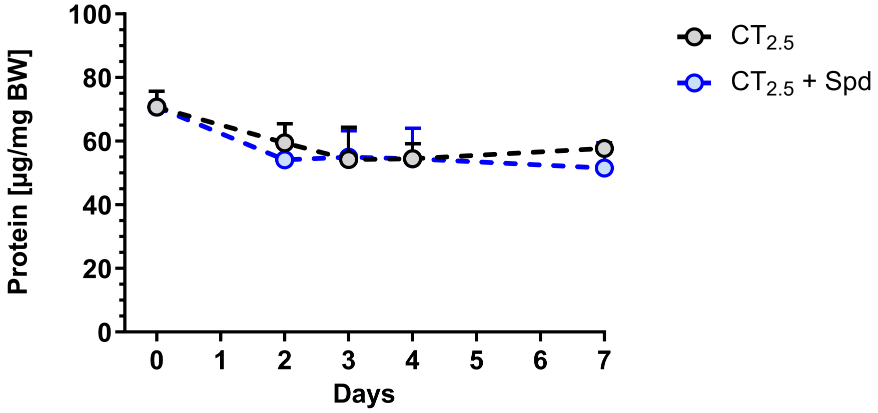

Fig. S1C

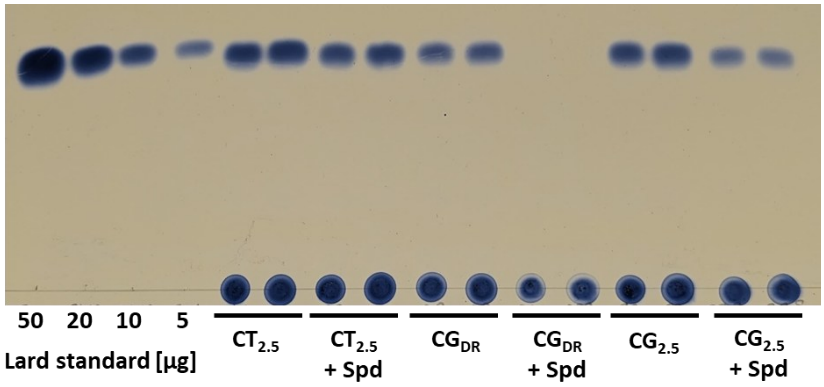

Fig. S1D

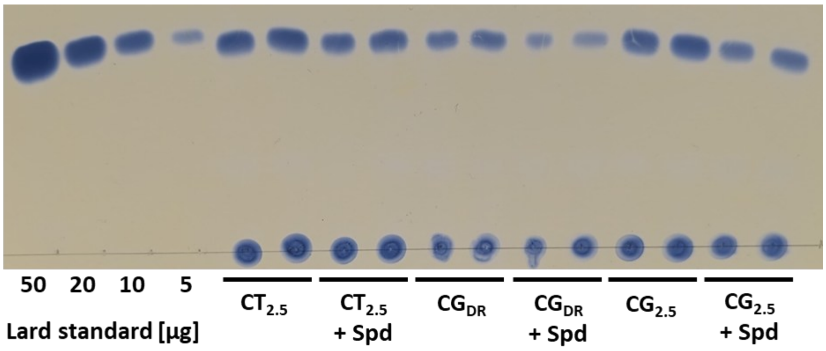

Fig S1E

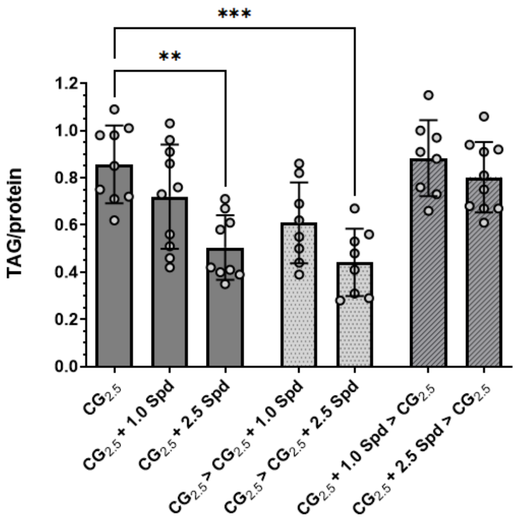

Fig. S1F

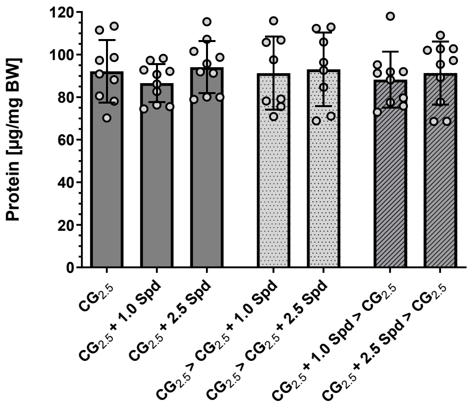

Fig. S1G-I

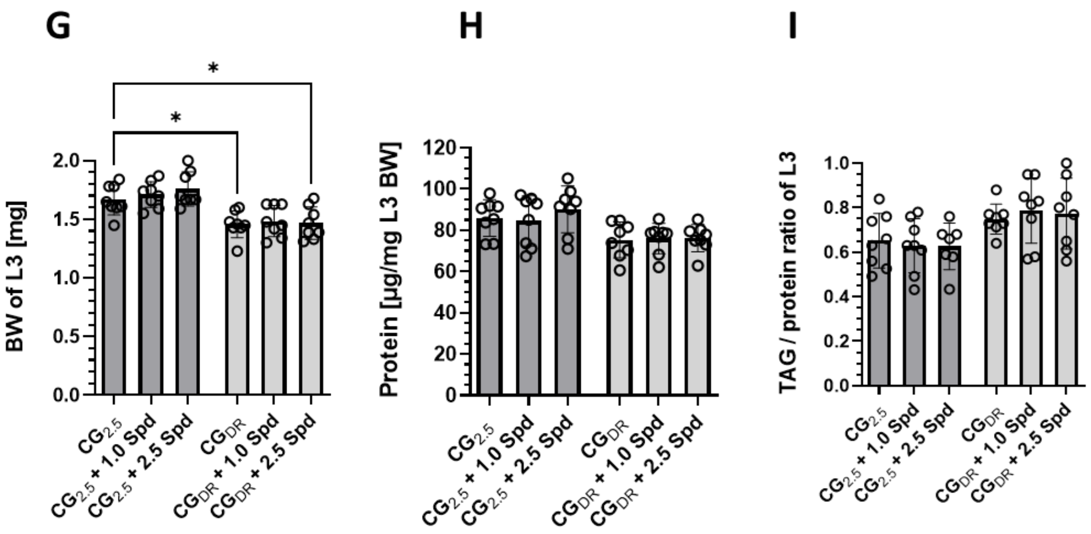

Fig. S1J, K

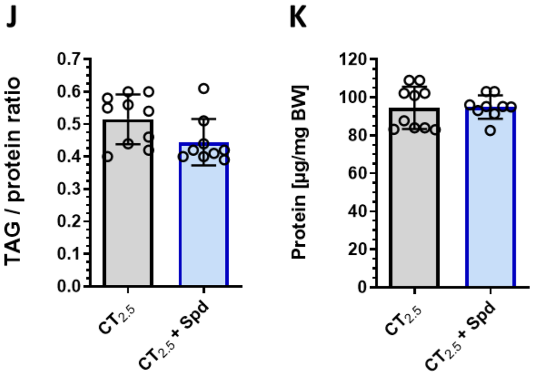

Fig. S1L

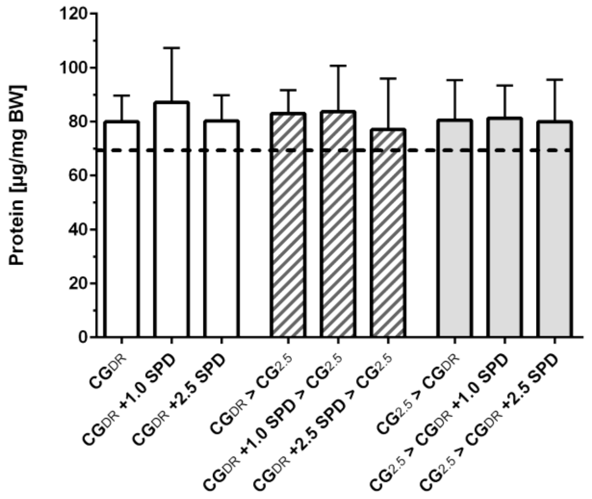

Fig. S1M

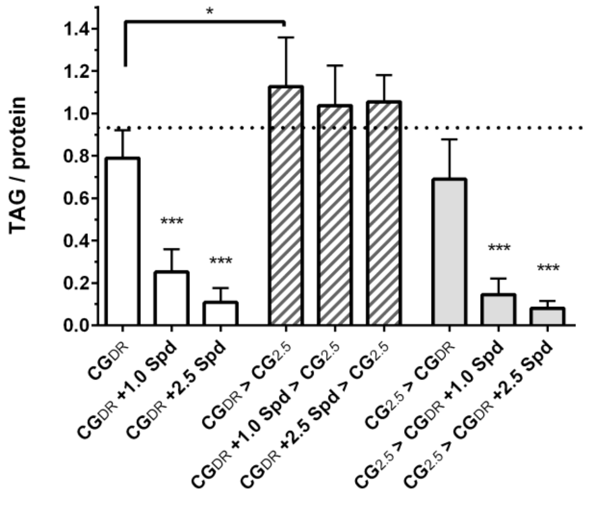

Figure S1. When $CT_{2.5}$ medium was employed, administration of spermidine prevented the accumulation of TAG stores in female fruit flies similarly to the data obtained with $CG_{2.5}$ medium.

(A, B) Freshly eclosed male and female *D. melanogaster* w^{1118} were transferred as mixed populations to the specified experimental diets. The animals were harvested at the time points indicated, sorted by sex and the protein content and the TAG-to-protein ratio of females were determined by colometric assays at the respective time points. Data points represent the mean of n = 6 experiments with N = 60 animals per condition and time point. For statistical analyses, two-way ANOVA followed by Sidak's multiple comparisons test was carried out. **(C)** The TAG lowering effect on w^{1118} female fruit flies achieved by

administering 2.5 mM spermidine (Spd) for a period of 7 days post-eclosion was confirmed by thin layer chromatography (TLC) analyses **(D)** and in a second *D. melanogaster* wild-type strain, Oregon RC. For the TLC analyses, lard was used as TAG standard at the indicated amounts. $CT_{2.5}$: Caltech medium; $CG_{2.5}$: cornmeal glucose control medium; CG_{DR}: dietary restriction medium. **(E, F)** Supplemented Spd (concentrations in in mM) had a dose-dependent TAG lowering effect on female w^{1118} fruit flies when present during the adult stage. The corresponding protein levels remain unaffected by any of the applied Spd treatment protocols. Synchronized eggs were allocated to the different experimental diets. If there was a medium change after the pupal stage, larval development took place on the diet indicated before the ">" symbol in the x-axis legend, while subsequent cultivation of freshly eclosed adult fruit flies occurred on the media named after the ">" symbol. On day 7, the flies were harvested and sorted by sex. The protein and TAG-to-protein ratios of females were determined. The experiment was conducted in n = 10 replicates with N = 100 animals per condition. Bars represent the mean ± standard deviation. Statistical significance was assumed at ** $p < 0.01$, *** $p < 0.001$ (evaluated by Kruskal-Wallis test followed by Dunn's multiple comparisons test). **(G-I)** The body composition of *D. melanogaster* w^{1118} larvae were affected by feeding a dietary restriction diet but not by the administration of Spd during larval development. Synchronized eggs were allocated to the different experimental diets. Wandering third instar larvae were harvested and their body weight and body composition were determined. Bars represent the mean ± standard deviation of eight replicates with N = 80 individuals. Statistical significance was assumed at * $p < 0.05$, ** $p < 0.01$ (Welch ANOVA followed by Dunnett's T3 multiple comparisons test (G, I) and Kruskal-Wallis test followed by Dunn's multiple comparisons test (H)). **(J, K)** The impact of Spd administration on male w^{1118} fruit flies fed a $CT_{2.5}$ diet. Freshly eclosed fruit flies were transferred as mixed populations to the specified experimental diets. On day 7, the animals were harvested, sorted by sex and the protein content and the TAG-to-protein ratio of males were determined by colometric assay. Bars represent the mean of n = 10 experiments with N = 100 animals per condition. A Mann-Whitney test was conducted for statistical analysis. **(L, M)** The impact of Spd administration during larval feeding with a dietary reduction diet (CG_{DR}) on the body composition of adult female *D. melanogaster*. Synchronized w^{1118} eggs were allocated to the indicated experimental diets, which

initially were all based on the CG_{DR} diet. Similar to (E, F), larval development occurred on the diet indicated prior to the “>” symbol in the x-axis legend, whereas subsequent cultivation of freshly eclosed adult fruit flies was conducted on the medium named after the “>” symbol, provided that there was a change in medium following the pupal stage. On day 7, the flies were harvested and sorted by sex. The protein and TAG-to-protein ratios of females were determined. The experiment was conducted in n = 10 replicates with N = 100 animals per condition. Bars represent the mean ± standard deviation. Statistical significance was assumed at * $p < 0.05$, *** $p < 0.001$ (evaluated by one-way ANOVA followed by Dunnett's multiple comparisons test).

Fig. S2A-E

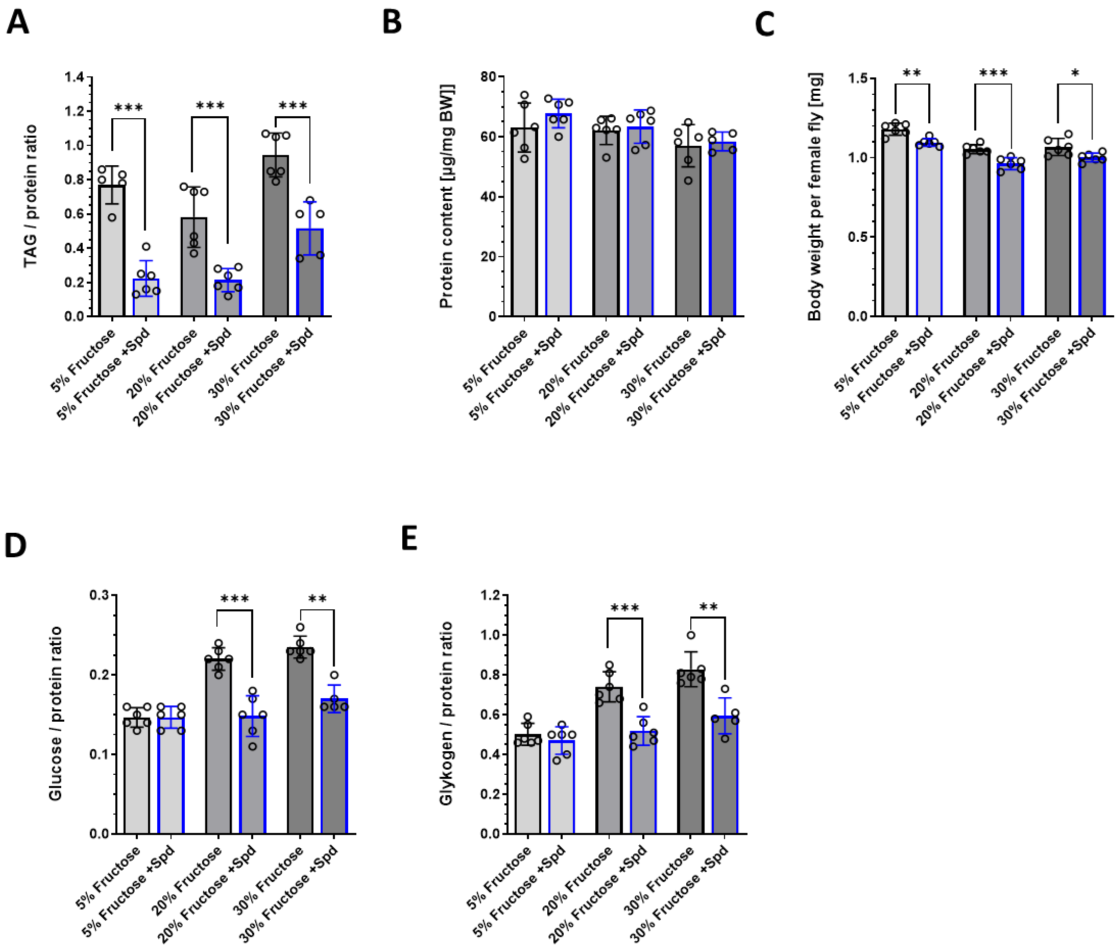
A
TAG / protein ratio
B
Protein content [µg/mg BW]]
C
Body weight per female fly [mg]
D
Glucose / protein ratio
E
Glykogen / protein ratio
5% Fructose
5% Fructose +Spd
20% Fructose
20% Fructose +Spd
30% Fructose
30% Fructose +Spd
***
**
*

Fig. S2F-J

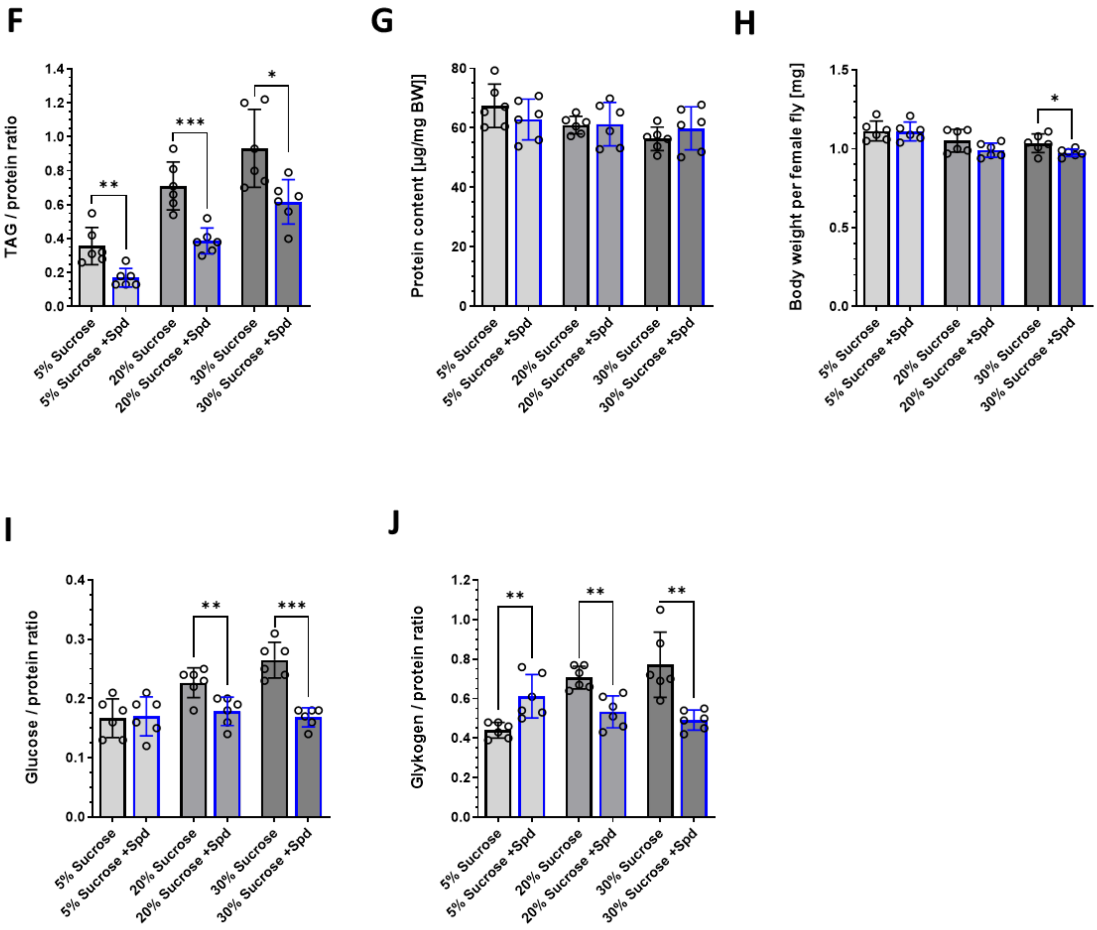

Figure S2 Spermidine supplementation counteracts a high-sugar diet-induced obesity in *D. melanogaster* females, irrespective of the type of sugar supplied. Corresponding to the data depicted in Figure 3, spermidine (Spd) given at 2.5 mM prevented the accumulation of TAG stores in female fruit flies when co-administered with a high-fructose **(A-E)** or high-sucrose diet **(F-J)**, which contained 2.5% inactive yeast extract. Freshly eclosed male and female fruit flies were transferred as mixed populations to the specified experimental diets. On day 7, the animals were harvested and sorted by sex. The body weight as well as the protein, TAG, glucose and glycogen content of females were determined. For these parameters, the effects of Spd supplementation were analysed in comparison to a corresponding control diet with the same sugar content. Data points represent the mean of n = 6 experiments with N = 60 animals per condition. Statistical significance was assumed at * $p < 0.05$, ** $p < 0.01$, *** $p < 0.001$ (evaluated by unpaired t-test or Mann-Whitney test).

Fig. S3A

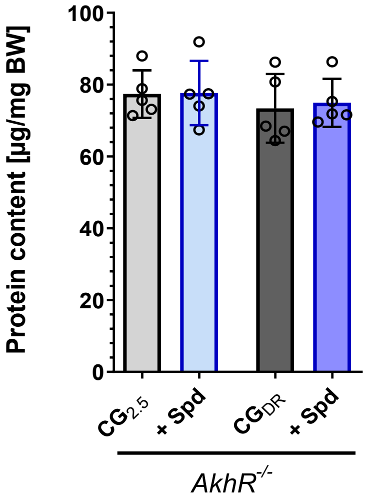

Fig. S3B

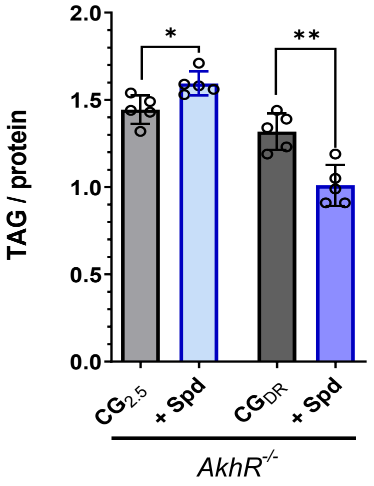

Figure S3 A significant TAG-reducing effect of spermidine administration occurs in *AkhR* loss-of-function mutants exposed to dietary restriction. (A, B) Freshly eclosed male and female *AkhR*$^{-/-}$ fruit flies were transferred as mixed populations to the specified experimental diets. On day 7, the animals were harvested and sorted by sex before the protein and TAG content of females were determined. Bars represent the mean of n = 5 experiments with N = 50 individuals per condition. Statistical analyses were performed using unpaired t-test to determine the impact of spermidine (Spd) supplementation on body composition (* $p < 0.05$; ** $p < 0.01$). *AkhR, Adipokinetic hormone receptor;* $CG_{2.5}$, cornmeal-glucose medium containing 2.5% inactive yeast extract; CG_{DR}, dietary restriction medium containing 50% cornmeal, glucose and inactive yeast extract of the $CG_{2.5}$ diet.

Fig. S4

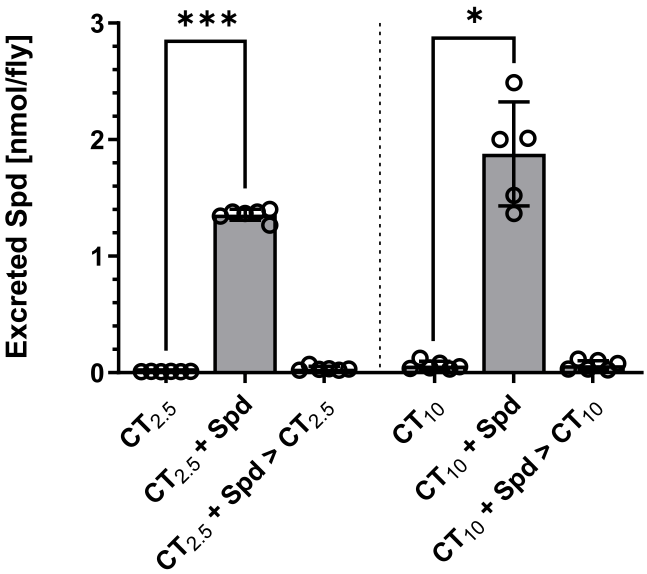

Figure S4 The predominant portion of dietary spermidine consumed by *D. melanogaster* is promptly excreted through the gastrointestinal tract. Freshly eclosed w^{1118} fruit flies were allocated in groups of 12 males and 12 females to the different experimental diets, namely $CT_{2.5}$ or CT_{10} or the corresponding media supplemented with 2.5 mM spermidine (Spd). On day 4, the pre-fed female flies were transferred to Ex-Q vials receiving either the same experimental diets or, where indicated by the ">" symbol, the respective diet without Spd supplementation. The flies were maintained for another 24 h, before they were removed on day 5. Subsequently, the excreta were harvested for HPLC analyses of the polyamine profile. $CT_{2.5}$, Caltech medium containing 2.5% inactive yeast extract; CT_{10}, Caltech medium containing 10% inactive yeast extract. Bars represent the mean SD of 6 independent experiments with N = 60 individuals per condition. Statistical analyses were performed using Kruskal-Wallis test followed by Dunn's multiple comparisons test (* $p < 0.05$; *** $p < 0.001$).
