# Supplementary material for: Homeostatic Counter‐Regulation Mediates Spermidine‐Induced Triacylglyceride Reduction in Drosophila melanogaster—From Phenotype to Molecular Mechanism
Source: FASEB J. 2025 Oct 21;39(20):e71153. doi: 10.1096/fj.202502620R (PMC12539389; doi:10.1096/fj.202502620R)
Supplement: Supplementary file 2 — Table S1: fsb271153‐sup‐0002‐TableS1.pdf. [file FSB2-39-e71153-s002.pdf]

Supplemental Tables

Table S1. The gradient used for separating dansylated polyamines by HPLC. The flow rate was 1.5 ml/min (Agilent 1100 HPLC system). Mobile phase A consists of 10 mM KH_2PO_4, pH 4.6 and mobile phase B of acetonitril:methanol (185:15, v/v). The gain of the fluorescence detector (FD gain) was adjusted to the abundance of the respective polyamines.

**Time [min]**	0	25	30	34	40	41	46
**% B**	35	60	64	90	90	35	35
**FD gain**	15	15	13	13	13	13	13

Table S2 Primer sequences used for quantitative RT-PCR. The following cycling conditions were applied: initial denaturation at 95°C for 10 s, followed by 40 cycles of amplification each including denaturation at 95°C for 5 s; annealing at 58°C for 10 s and extension at 72°C for 5 s. F = forward primer; R = reverse primer. CG4210 is the annotation symbol (https://flybase.org/) for the *Sat* gene, CG8032 for the *pao* gene.

**Primer**	**5' > 3' sequence**
Dm rpl32-F	GGCAAGCTTCAAGATGACCA
Dm rpl32-R	GTTCGATCCGTAACCGATGT
Dm CG4210-F	AGATGTGCTCTCCATGATTCAAGA
Dm CG4210-R	GTGAGTCCTGCGTCTCGTTTA
Dm CG8032-F	GCGGACGCATTGTGTCCATA
Dm CG8032-R	CAGAACGCCGTGTATCCAGTT
Dm acc-F	GGCGACAACCTTGTAGAGAT
Dm acc-R	CAAGTAGGTGAGATCGTTGG
Dm bmm-F	AGTATGCACCGCATCTGTTG
Dm bmm-R	TGGATGTTGAAGGAGGGACT

Dm hsl-F	GCAGTCCTACGAGATTCACG
Dm hsl-R	GGTCCATGTTAAGTGTAAGTATTTTGG
Dm mdy-F	GTCTTCCATGAATATTTGGTTTCAG
Dm mdy-R	CCA TTA TGC ACA GAG GCT GA

Tab. S3 Statistics related to Figure 8B. A two-way ANOVA analysis was conducted followed by a Sidak's multiple comparisons test with *p < 0.05, ***p < 0.001 and ns = not significant. N1AcSpd, N^1-acetylspermidine; Put, putrescine.

**Put**	
$CT_{2.5}$ vs. $CT_{2.5}$ +2.5 Spd	***
$CT_{2.5}$ vs. CT_{10}	*
$CT_{2.5}$ vs. CT_{10} +2.5 Spd	***
$CT_{2.5}$ +2.5 Spd vs. CT_{10}	***
$CT_{2.5}$ +2.5 Spd vs. CT_{10} +2.5 Spd	***
CT_{10} vs. CT_{10} +2.5 Spd	***

**N1AcSpd**	
$CT_{2.5}$ vs. $CT_{2.5}$ +2.5 Spd	***
$CT_{2.5}$ vs. CT_{10}	ns
$CT_{2.5}$ vs. CT_{10} +2.5 Spd	***
$CT_{2.5}$ +2.5 Spd vs. CT_{10}	***
$CT_{2.5}$ +2.5 Spd vs. CT_{10} +2.5 Spd	***
CT_{10} vs. CT_{10} +2.5 Spd	***

Tab. S4 Statistics related to Figure 8C. A two-way ANOVA analysis was conducted followed by a Sidak's multiple comparisons test with ***p < 0.001 and ns = not significant. $AkhR^{-/-}$, Adipokinetic hormone Receptor; N1AcSpd, N^1-acetylspermidine; Put, putrescine.

**Put**	
w^{1118} $CT_{2.5}$ vs. w^{1118} $CT_{2.5}$ +2.5 Spd	***
w^{1118} $CT_{2.5}$ vs. $AkhR^{-/-}$ $CT_{2.5}$	ns
w^{1118} $CT_{2.5}$ vs. $AkhR^{-/-}$ $CT_{2.5}$ +2.5 Spd	***
w^{1118} $CT_{2.5}$ +2.5 Spd vs. $AkhR^{-/-}$ $CT_{2.5}$	***
w^{1118} $CT_{2.5}$ +2.5 Spd vs. $AkhR^{-/-}$ $CT_{2.5}$ +2.5 Spd	ns
$AkhR^{-/-}$ $CT_{2.5}$ vs. $AkhR^{-/-}$ $CT_{2.5}$ +2.5 Spd	***
**N1AcSPD**	
w^{1118} $CT_{2.5}$ vs. w^{1118} $CT_{2.5}$ +2.5 Spd	***
w^{1118} $CT_{2.5}$ vs. $AkhR^{-/-}$ $CT_{2.5}$	ns
w^{1118} $CT_{2.5}$ vs $AkhR^{-/-}$ $CT_{2.5}$ +2.5 Spd	***
w^{1118} $CT_{2.5}$ +2.5 Spd vs. $AkhR^{-/-}$ $CT_{2.5}$	***
w^{1118} $CT_{2.5}$ +2.5 Spd vs $AkhR^{-/-}$ $CT_{2.5}$ +2.5 Spd	***
$AkhR^{-/-}$ $CT_{2.5}$ vs. $AkhR^{-/-}$ $CT_{2.5}$ +2.5 Spd	***
